# Traditional Chinese Patent Medicine for Primary Hypertension: A Bayesian Network Meta-Analysis

**DOI:** 10.1155/2020/6701272

**Published:** 2020-04-24

**Authors:** Zhe Chen, Qingyang Shi, Lizi Tan, Yingying Peng, Chunxiang Liu, Junhua Zhang

**Affiliations:** ^1^Tianjin University of Traditional Chinese Medicine, Tianjin 301617, China; ^2^Evidence-based Medicine Center, Tianjin University of Traditional Chinese Medicine, Tianjin 301617, China

## Abstract

**Background:**

Traditional Chinese Patent Medicine (TCPM) is now being used more and more extensively for primary hypertension in China. However, the comparative efficacy and safety of it need more clarified evidence. Thus, we conducted a Bayesian network meta-analysis to compare TCPMs with other interventions.

**Methods:**

We searched China National Knowledge Infrastructure (CNKI), WanFang Data, PubMed, Embase, and Cochrane Library from inception to April 2019 for randomized controlled trials (RCTs) with diagnosis of primary hypertension that compared the efficacy of TCPMs with antihypertension drugs (ADs). Two researchers screened literature, extracted data, and evaluated risk of bias independently. The primary outcomes were systolic blood pressure (SBP) and diastolic blood pressure (DBP). The secondary outcomes were adverse effects (AEs), total cholesterol (TC), and triglyceride (TG). We used the Bayesian network meta-analysis to compare interventions and described the categorical variable and the continuous variable as odds ratio (OR) and mean difference (MD), respectively. Besides, we ranked all interventions via the Surface Under the Cumulative Ranking (SUCRA) values and conducted metaregression with nine covariates as additional analysis.

**Results:**

We included 192 studies with 23366 patients diagnosed as primary hypertension in total. For SBP reduction, eighteen interventions were significantly better than AD. Among them, Yinxingye (YXY) + AD (MD = −12, 95% CrI [−16, −8.5]) was superior to others in the rank plot with SUCRA 0.91. For DBP reduction, sixteen interventions were significantly better than AD. Among them, Qinggan Jiangya (QGJY) + AD (MD = −8.7, 95% CrI [−12, −5.5]) and Qiju Dihuang (QJDH) + AD (MD = −8.8, 95% CrI [−12, −5.2]) were superior to others in the rank plot with SUCRA 0.89. To summarize the SUCRA values, we found that QGJY + AD and YXY + AD had the most significant reductions for both SBP and DBP. YXY + AD was the best one for both TC (MD = −1.3, 95% CrI [−1.9, −0.64]) and TG (MD = −0.52, 95% CrI [−0.92, −0.11]) reductions. Considering adverse effects, we found two interventions had significant differences comparing with AD. Among them, YXY + AD was the best one with SUCRA of 0.01.

**Conclusion:**

In all TCPMs, QGJY + AD and YXY + AD may be the best options for hypertension. Meanwhile, YXY + AD can improve blood lipids in patients with hypertension. However, due to the vague reports of adverse effects and other limitations, more evidence, especially that provided by high-quality studies, is needed to prove the advantages of TCMPs.

## 1. Introduction

Hypertension is a worldwide disease and is one of the important risk factors for cardiovascular and kidney diseases [1]. With the acceleration of population aging and the rising of population exposure risks, the global incidence and prevalence of hypertension are increasing annually [2]. As estimated, the number of patients with hypertension is expected to reach 1.56 billion by 2025 [3]. There exists no difference between genders when considering this high incidence of hypertension, but the overall incidence has a positive correlation with age [3]. Due to the different situations in each country's economic and medical care, there are great differences in the prevention, diagnosis, and treatment of hypertension worldwide, and the incidence of less developed countries has increased significantly [1, 4, 5].

Being the most populous developing country in the world, China's hypertension incidence is at a high level [6]. The diagnose boundary values in China are defined as 140 mmHg for systolic blood pressure (SBP) and 90 mmHg for diastolic blood pressure (DBP) which is different from that made by American College of Cardiology (ACC) as SBP ≥130 mmHg or DBP ≥80 mmHg [7–9]. A study showed that about 42.7% of Chinese hypertensive patients understand their health problem. However, only 8.3% of Chinese hypertension patients have been under control [10]. According to several studies, family genetics [11], environment [12], overweight or obesity [13], high salt diet [14, 15], smoking [16], and insobriety [17] are the main risk factors for hypertension. China is now facing a high prevalence of high blood pressure caused by various risk factors [18].

Hypertension is closely linked to cardiovascular disease [19], diabetes [20], and kidney disease [21]. By 2030, the estimated number of global deaths due to cardiovascular disease caused by high blood pressure will surge, and the annual death toll is expected to reach 23.6 million [22]. A study shows that three-quarters of diabetic patients tend to have high blood pressure, and patients with both high blood pressure and diabetes have a much higher probability of developing cardiovascular disease and a fourfold increase in mortality [23]. When the renal resistive index of patients with kidney disease caused by essential hypertension is greater than 0.7, the mortality rate will increase [24], which indicates the impact of hypertension on kidney disease patients.

Currently, the main antihypertensive drugs (ADs) commonly used in clinical practice include calcium channel blockers (CCB), angiotensin-converting enzyme inhibitor (ACEI), angiotensin-receptor blockers (ARB), diuretics, and beta-blockers (BB) [25]. Treatment of hypertension often uses both monotherapy and combination therapy [26]. Although the treatment of ADs is effective in short term, it will bring drug resistance and side effects in long term [27]. Therefore, many researchers have explored the field of complementary and alternative medicines to find new methods to deal with this challenge. Traditional Chinese medicine (TCM) has a long history in clinical practice and can effectively and safely prevent and treat some diseases, which made it an important component in complementary and alternative medicines [28, 29]. A geriatric cohort study on Taiwan shows that about half of the participants have received TCM for treatment [29]. According to a survey conducted in mainland China, more than 90% of respondents believe that integration of TCM and modern western medicine is the best diagnosis and treatment method [30]. According to the statistical data revealed from the National Bureau of Statistics of China in 2011, the industrial output value related to TCM reached 68 billion in US dollars, which increased 37.9% compared with last year [11].

The combination of TCM and ADs in the treatment of essential hypertension can control blood pressure and protect target organs and also improve patients' life qualities and clinical symptoms [31, 32]. As a kind of TCM product with national quality standards, traditional Chinese patent medicine (TCPM) has been registered and sold in various countries around the world [33]. The antihypertensive effect of TCPMs has been proven to be safe and effective, which made it a good substitute for western medicine intolerance patients [34]. Cardiovascular experts have reached a consensus on the treatment of hypertension with TCPMs. They believe that TCPMs and ADs can complement each other's disadvantages, and the combination of these two interventions can effectively stabilize blood pressure and improve clinical symptoms [35]. Several studies have shown that TCPMs has a good blood pressure controlling effect. In addition, TCPMs performed a good role in dealing with inflammation, endothelial dysfunction, and dyslipidemia, and it can promote nerve regeneration and angiogenesis, which will provide protection to the heart and related organs [36–38]. Some meta-analysis results indicated that TCPMs combined with ADs is superior to ADs alone, which can effectively improve blood pressure and patients' life qualities [39–41].

At present, the integration of TCPMs and ADs has become a new intervention for hypertension. One review [39] has compared some TCPMs in treating EH; however, it failed to provide conclusions on the rank of interventions considering efficacy and safety due to a small number of included studies and limitations of its analysis method. Therefore, this study used a network analysis method to evaluate the clinical efficacy and safety of different TCPMs in the treatment of EH in order to provide some evidence for clinical decision-making.

## 2. Methods

We performed the systematic review and network meta-analysis following the reporting standards guidance from the PRISMA and its extension statement for network meta-analysis [42, 43]. We also followed our protocol that was registered in PROSPERO with ID CRD42019134646. In addition, we did some amendments to the protocol, and details were shown in S1 File 1.

### 2.1. Included and Excluded Criteria

We included randomized controlled studies with a diagnosis of primary hypertension, regardless of whether it had comorbidity or not. Treatments were limited to oral TCPMs that were approved by the Chinese State Drug Administration. The treatments of interest were as follows: oral TCPMs combined with conventional ADs versus conventional ADs or oral TCPMs versus conventional ADs or contrast between different types of oral TCPMs. In addition, TCPM, which was searched with less than three clinical trials, was excluded. The types of AD were not restricted. All clinical trials must report both DBP and SBP in baseline and follow-up endpoint, otherwise, be excluded. We excluded secondary hypertension, severe injury of organ function, systematic review, and animal experiment.

### 2.2. Literature Search

The data sources included China National Knowledge Infrastructure (CNKI), WanFang Data, PubMed, Embase, and Cochrane Library. Main search terms were “Randomized controlled trial (RCT)”, “Primary hypertension” and “Traditional Chinese patent medicine” with a combination of MeSH and free terms. The detailed search strategy was shown in the supplementary appendix (S1 File 2). We searched these databases from inception to April 2019, exported citations using Endnote, and removed duplicates. Besides, we also searched references from relevant meta-analysis in case of any omissive trials.

### 2.3. Literature Screening and Data Extraction

The screening process was conducted by two independent researchers with cross check. Records and abstracts were screened first, and then full-text articles were screened after we excluded irrelevant articles. Disagreements were judged by another researcher. The information of extraction was as follows: study characters: author, years of publication, count of total participant, treatment duration; participant characters: age, gender, course of disease, comorbidity or not; interventions and comparisons: types of TCPMs, types of ADs; outcomes.

### 2.4. Risk of Bias Assessment

Two researchers independently assessed the risk of bias using Cochrane risk of bias tool. The seven assessed terms were as follows: random sequence generation; allocation concealment; blinding of participants and personnel; blinding of outcome assessment; incomplete outcome data; selective reporting; other bias. Disagreements were solved by the third researcher [44].

### 2.5. Outcome Measurements

Primary outcomes: systolic blood pressure (SBP); diastolic blood pressure (DBP).

Secondary outcomes: total cholesterol (TC), triglyceride (TG), and adverse effects (AEs).

### 2.6. Statistical Analysis

A Bayesian network meta-analysis was conducted with a random effect model to synthesize the data for each outcome. The model was based on consistency assumption between direct and indirect comparisons. Moreover, we used a vague prior distribution for all estimations and set a parameter *σ*∼Unif (0, *N*) for between-study heterogeneity. The posterior estimations were obtained using Markov Chain Monte Carlo (MCMC) method. Furthermore, we used the Brooks-Gelman-Rubin method to detect the convergence of the model. The categorical and continuous variables were described using (Log) odd ratio (OR) and mean difference (MD), respectively. The between-study heterogeneity was quantitated through I-square. If any loop of three interventions existed, we used the node-splitting method to present both direct and indirect results with inconsistent *P* values.

The median of posterior distribution and the corresponding 95% credible interval were calculated and presented as the relative effects of outcomes. Moreover, we ranked all interventions by their posterior probability via the Surface Under the Cumulative Ranking (SUCRA) curve values. The small-study effects and the publication bias were detected by the comparison-adjusted funnel plots with the specific ranking order.

All the analyses were done using *R* 3.6.0 along with the Markov Chain Monte Carle engine JAGS 3.4.0, and the risk of bias graphs was performed by the Cochrane tool RevMan 5.3.

## 3. Result

### 3.1. Literature Review

**Figure 1:**
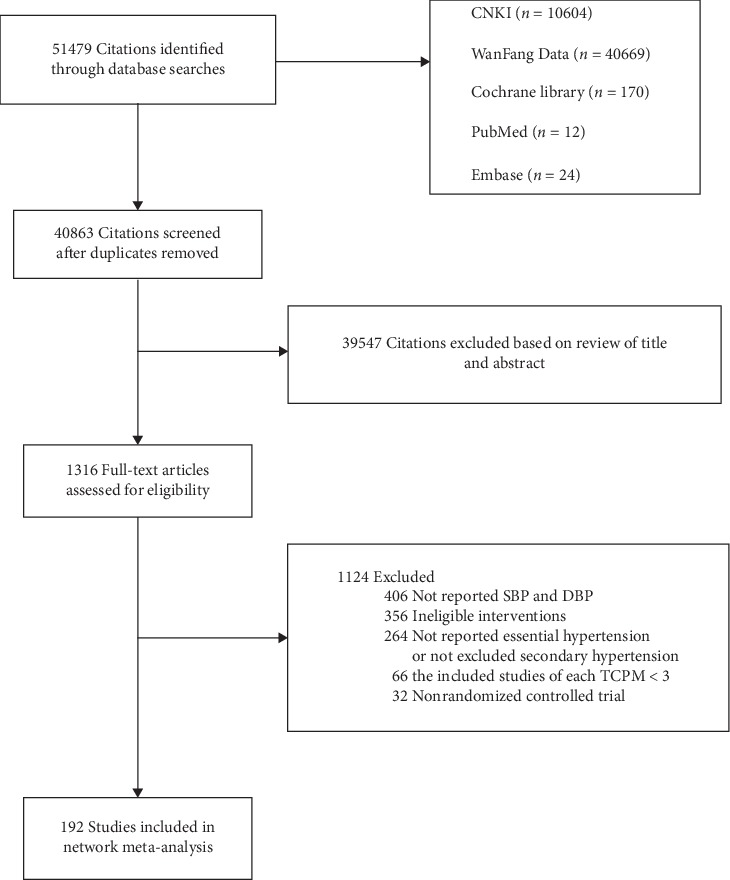
Summary of evidence search and selection.

Of 51479 studies identified, 1316 studies were moved into potential full-text reading after exclusion of duplicates and ineligible articles through screening titles and abstracts. Then, we excluded studies in the literature that did not meet the criteria and finally included 192 RCTs in the analysis (Figure 1).

We included 192 studies (S1 Table 1) in total involving 23366 patients diagnosed with primary hypertension. Of them, the proportion of males was 50.3%, the median age was 58 years with a range of 18–80, the median duration of treatment was 8 weeks with a range of 4 weeks to 2 years, and median course of the disease was 7.6 years with a range of 1–20 years. Seventy-six studies reported comorbidities, including 43 studies with cardiovascular disease, 11 with kidney disease, 28 with diabetes, and 17 with hyperlipemia.

All interventions were classified into 27 categories. Twenty-two of them were combinations of TCPMs and ADs, 4 were TCPMs alone, and 1 was ADs alone. TCPMs included Shensong Yangxin capsule (SSYX), Jinshuibao capsule (JSB), Naoxintong capsule (NXT), Qinggan Jiangya capsule (QGJY), Quantianma capsule (QTM), Songlin Xuemaikang capsule (SLXMK), Tongxinluo capsule (TXL), Wuling capsule (WL), Xuemaitong capsule (XMT), Xuezhikang capsule (XZK), Yindan Xinnaotong capsule (YDXNT), Tianma Gouteng particle (TMGT), Wenxin particle (WX), Yangxue Qingnao particle (YXQN), Qiangli Dingxuan tablet (QLDX), Xinkeshu tablet (XKS), Yinxingye dropping pill or tablet (YXY), zhenju Jiangya tablet (ZJJY), Fufang Danshen dropping pill (FFDS), Liuwei Dihuang pill (LWDH), Niuhuang Jiangya pill (NHJY), Qiju Dihuang pill (QJDH), and Shexiang Baoxin pill (SXBX). AD included Calcium channel blocker (CCB), Angiotensin-converting enzyme inhibitor (ACEI), Angiotensin II receptor antagonist (ARB), diuretics, and Beta-blocker (BB).

All studies reported both SBP and DBP with a changed amount of baseline to endpoint. Eighty-one studies reported adverse events which included gastrointestinal system (i.e., nausea, emesis, diarrhea, and constipation), headache, dizziness, facial flush, skin disease (Pruritus, rash), edema, cough, hypotension, and so on. Furthermore, 46 studies reported a number of subjects with adverse events. Thirty-six studies reported total cholesterol of before and after treatment, and 29 studies reported triglyceride (S2 Table 2).

Of included 192 studies, all of them reported “random” but only 33 studies reported methods of random sequence generation, 1 study reported allocation concealment, 18 reported double-blind, and 41 studies existed selective reporting (S2 Figure 1).

### 3.2. Network Meta-Analysis

#### 3.2.1. Primary Outcomes

**Figure 2:**
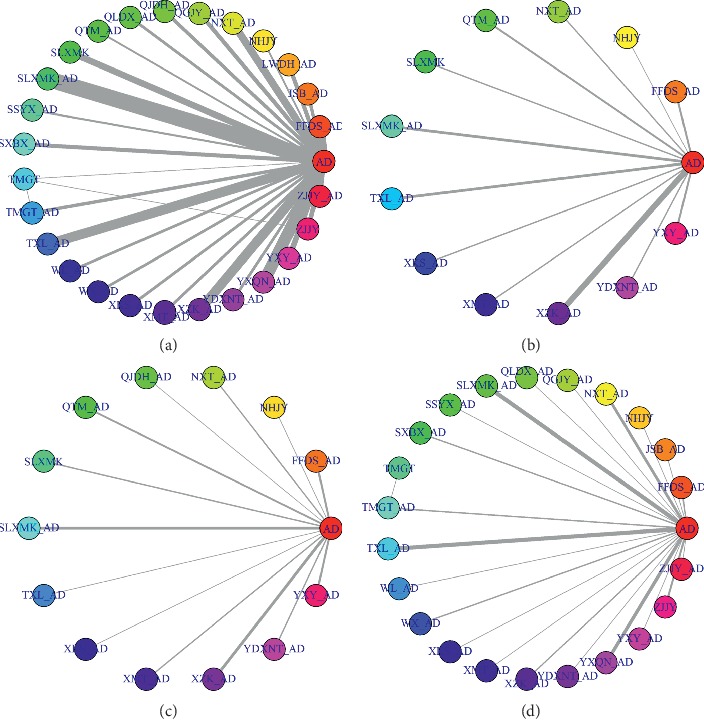
Network plots. (a) Systolic blood pressure and diastolic blood pressure. (b) Total cholesterol. (c) Triglyceride. (d) Adverse effects. AD: antihypertensive drugs; SSYX: Shensong Yangxin capsule; JSB: Jinshuibao capsule; NXT: Naoxintong capsule; QGJY: Qinggan Jiangya capsule; QTM: Quantianma capsule; SLXMK: Songlin Xuemaikang capsule; TXL: Tongxinluo capsule; WL: Wuling capsule; XMT: Xuemaitong capsule; XZK: Xuezhikang capsule; YDXNT: Yindan Xinnaotong capsule; TMGT: Tianma Gouteng particle; WX: Wenxin particle; YXQN: Yangxue Qingnao particle; QLDX: Qiangli Dingxuan tablet; XKS: Xinkeshu tablet; YXY: Yinxingye dropping pill or tablet; ZJJY: zhenju Jiangya tablet; FFDS: Fufang Danshen dropping pill; LWDH: Liuwei Dihuang pill; NHJY: Niuhuang Jiangya pill; QJDH: Qiju Dihuang pill; (SXBX) Shexiang Baoxin pill.

**Figure 3:**
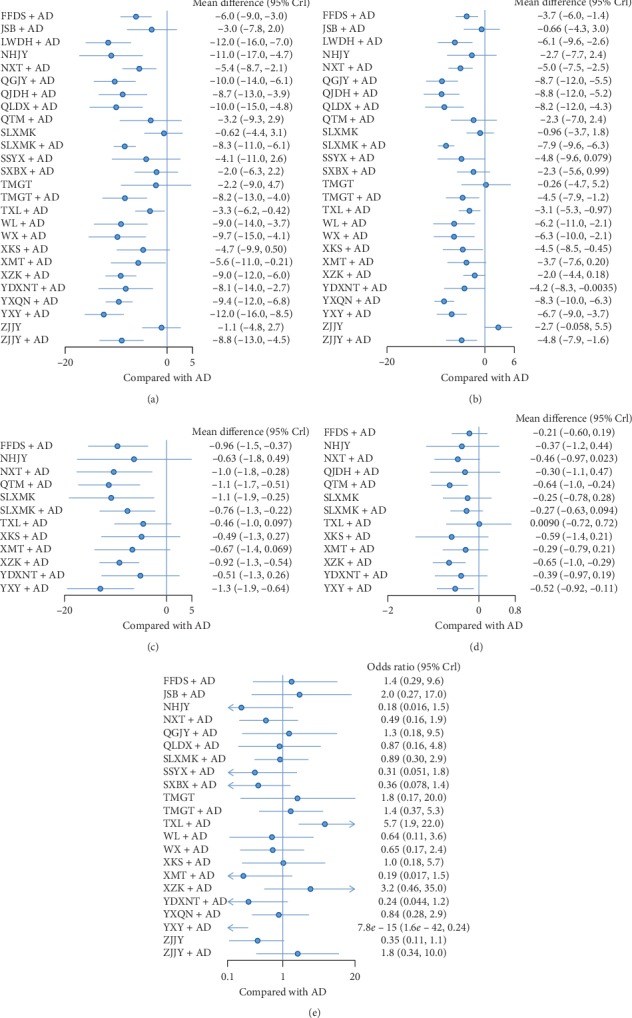
Forest Plots. (a) Systolic blood pressure. (b) Diastolic blood pressure. (c) Total cholesterol. (d) Triglyceride. (e) Adverse effects.

**Figure 4:**
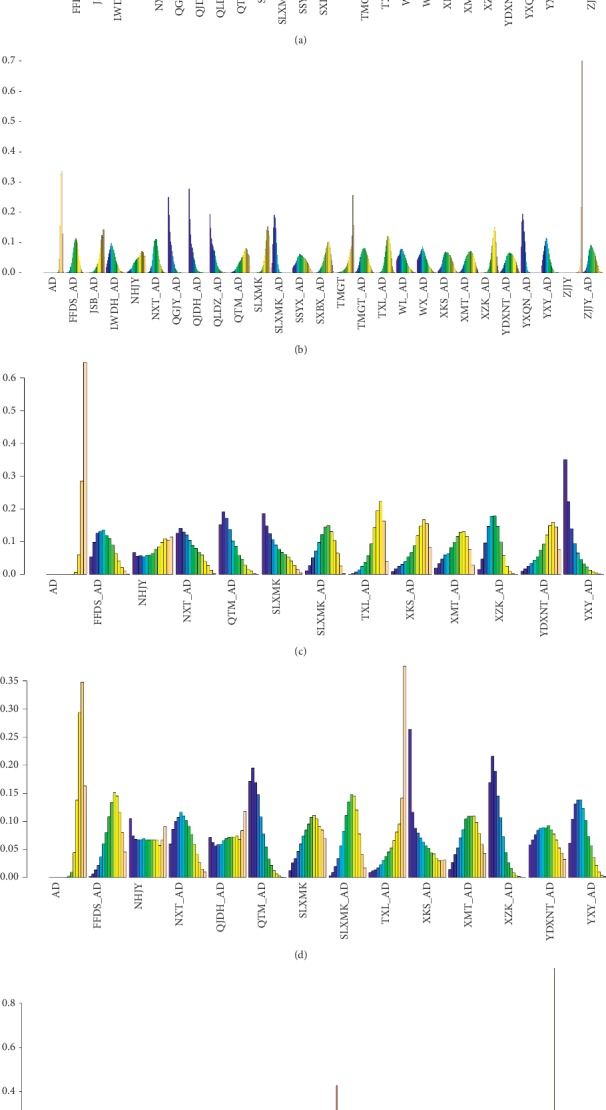
Rank Plots. (a) Systolic blood pressure. (b) Diastolic blood pressure. (c) Total cholesterol. (d) Triglyceride. (e) Adverse effects.

*(1) Systolic Blood Pressure*. All studies reported SBP in primary outcomes, involving 27 interventions. The network plot is shown in Figure 2(a). Sixteen interventions had a significant difference compared with AD alone (Figure 3(a)). (FFDS + AD : MD = −6.0, 95% CrI [−9.0, −3.0]; LWDH + AD : MD = −12, 95% CrI [−16, −7]; NHJY : MD = −11, 95% CrI [−17, −4.7]; NXT + AD : MD = −5.4, 95% CrI [−8.7, −2.1]; QGJY + AD : MD = −10, 95% CrI [−14, −6.1]; QJDH + AD : MD = −8.7, 95% CrI [−13, −3.9]; QLDX + AD : MD = −10, 95% CrI [−15, −4.8]; SLXMK + AD : MD = −8.3, 95% CrI [−11, −6.1]; TMGT + AD : MD = −8.2, 95% CrI [−13, −4]; TXL + AD : MD = −3.3, 95% CrI [−6.2, −0.42]; WL + AD : MD = −9.0, 95% CrI [−14, −3.7]; WX + AD : MD = −9.7, 95% CrI [−15, −4.1]; XMT + AD : MD = −5.6, 95% CrI [−11, −0.21]; XZK + AD : MD = −9.0, 95% CrI [−12, −6]; YDXNT + AD : MD = −8.1, 95% CrI [−14, −2.7]; YXQN + AD : MD = −9.4, 95% CrI [−12, −6.8]; YXY + AD : MD = −12, 95% CrI [−16, −8.5]; ZJJY + AD : MD = −8.8, 95% CrI [−13, −4.5]). The network results compared with each other are shown in Table 1. In the rank plot (Figure 4(a)), we found that YXY + AD was more effective than others, followed by LWDH + AD, NHJY, QGJY + AD and QLDX + AD, with 0.91, 0.86, 0.8, 0.78, and 0.75 SUCRA values, respectively (Table 2). In the node-splitting analysis, no inconsistency was detected between direct and indirect results (S2 Figure 2(a)). Furthermore, no significant coefficient was found for nine covariates in the metaregression (S1).

*(2) Diastolic Blood Pressure*. All studies reported DBP in primary outcomes, involving 27 interventions, which was the same as that of SBP. The network plot was shown in Figure 2(a). Fifteen interventions were significantly different when compared with AD alone (Figure 3(b)). The network results which are compared with each other are shown in Table 1. (FFDS + AD : MD = −3.7, 95% CrI [−6, −1.4]; LWDH + AD : MD = −6.1, 95% CrI [−9.6, −2.6]; NXT + AD : MD = −5.0, 95% CrI [−7.5, −2.5]; QGJY + AD : MD = −8.7, 95% CrI [−12, −5.5]; QJDH + AD : MD = −8.8, 95% CrI [−12, −5.2]; QLDX + AD : MD = −8.2, 95% CrI [−12, −4.3]; SLXMK + AD : MD = −7.9, 95% CrI [−9.6, −6.3]; TMGT + AD : MD = −4.5, 95% CrI [−7.9, −1.2]; TXL + AD : MD = −3.1, 95% CrI [−5.3, −0.97]; WL + AD : MD = −6.2, 95% CrI [−11, −2.1]; WX + AD : MD = −6.3, 95% CrI [−10, −2.1]; XKS + AD : MD = −4.5, 95% CrI [−8.5, −0.45]; YDXNT + AD : MD = −4.2, 95% CrI [−8.3, −0.0035]; YXQN + AD : MD = −8.3, 95% CrI [−10, −6.3]; YXY + AD : MD = −6.7, 95% CrI [−9.8, −3.7]; ZJJY + AD : MD = −4.8, 95% CrI [−7.9, −1.6]). In the rank plot (Figure 4(b)), QGJY + AD and QJDH + AD showed the same top efficacy compared with others, and these two were followed by YXQN + AD, QLDX + AD, and SLXMK + AD with tiny differences. In the node-splitting analysis, we found no significant differences (S2 Figure 2(b)). In the metaregression, we detected only one significant coefficient (i.e., comorbidity or not), in which 95% CrI did not include zero (*β* = −2.1371, 95% CrI [−3.53, −0.72]) (S1 Table 3). Moreover, after adjustment, we found that some SUCRA values changed significantly; among them we noticed that the SUCRA value of QJDH + AD raised into the top one. The other changed values are shown in Table 1.

**Figure 5:**
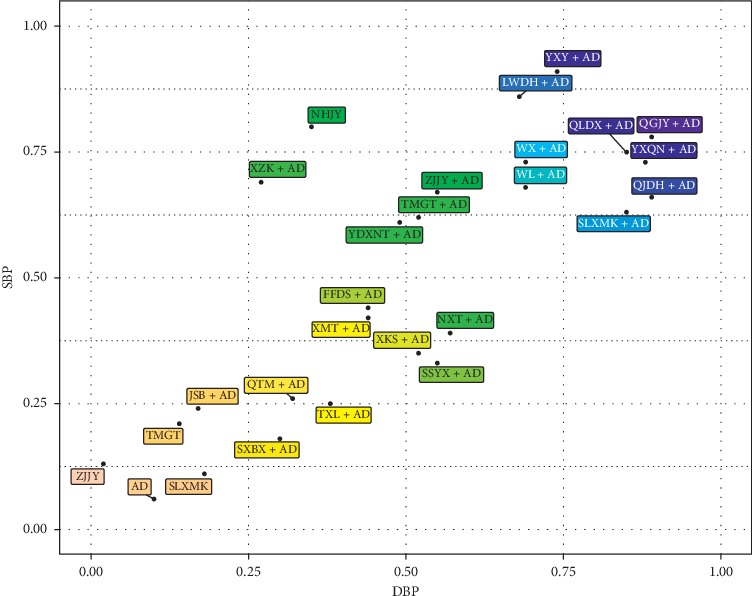
SUCRA biplot of Systolic blood pressure (SBP) and diastolic blood pressure (DBP).

After summarizing the SUCRA values of SBP and DBP in Figure 5, we found QGJY + AD was the only one with a greater SUCRA value than 0.75 in both outcomes, which might be the best one in primary outcomes, and followed by QGJY + AD, QLDX + AD, YXY + AD, YXQN + AD, QJDH + AD, LWDH + AD, WX + AD and WL + AD, whose SCURA values were greater than 0.6 in both outcomes.

#### 3.2.2. Secondary Outcomes

*(1) Total Cholesterol*. Including 13 interventions, 36 studies reported TC as a secondary outcome and the details are shown in Figure 2(b). Seven interventions were significantly different when compared with AD (Figure 3(c)). (FFDS + AD : MD = −0.96, 95% CrI [−1.5, −0.37]; NXT + AD : MD = −1.0, 95% CrI [−1.8, −0.28]; QTM + AD : MD = −1.1, 95% CrI [−1.7, −0.51]; SLXMK : MD = −1.1, 95% CrI [−1.9, −0.25]; SLXMK + AD : MD = −0.76, 95% CrI [−1.3, −0.22]; XZK + AD : MD = −0.92, 95% CrI [−1.3, −0.54]; YXY + AD : MD = −1.3, 95% CrI [−1.9, −0.64]). YXY + AD was superior to others in the rank plot (Figure 4(c)), followed by QTM + AD, SLXMK, NXT + AD, and FFDS + AD, with 0.84, 0.75, 0.7, 0.68 and 0.64 SUCRA, respectively (Table 2).

*(2) Triglyceride*. 29 studies reported TG and 14 interventions were included (Figure 2(c)). Three interventions were significantly different when compared with AD (Figure 3(d)). (QTM + AD : MD = −0.64, 95% CrI [−1.0, −0.24]; XZK + AD : MD = −0.65, 95% CrI [−1.0, −0.29]; YXY + AD : MD = −0.52, 95% CrI [−0.92, −0.11]). Moreover, in the rank plot (Figure 4(d)), XZK + AD showed the best efficacy than others, followed by QTM + AD, with SUCRA 0.81 and 0.79, respectively (Table 2).

*(3) Adverse Effects*. 46 studies reported numbers of subjects with adverse effects, including 24 interventions (Figure 2(d)). It showed that two interventions had significant difference compared with AD (Figure 3(e)). Among them, YXY + AD had lower risk than AD and TXL + AD had higher risk than AD. In the rank plot (Figure 4(e)), YXY + AD was significantly better than others with SUCRA 0.01 (Table 2).

In addition, 81 studies reported 676 adverse events, including 227 events of using TCPM combined with AD, 52 events of TCPM alone and 397 events of AD alone, whose details are shown in S1 Table 2. For the gastrointestinal system, events of TCPM combined with AD treatment were more than AD alone, especially SLXMK and TXL. For others, events of combination treatment were less than AD alone (S1 Table 2).

**Figure 6:**
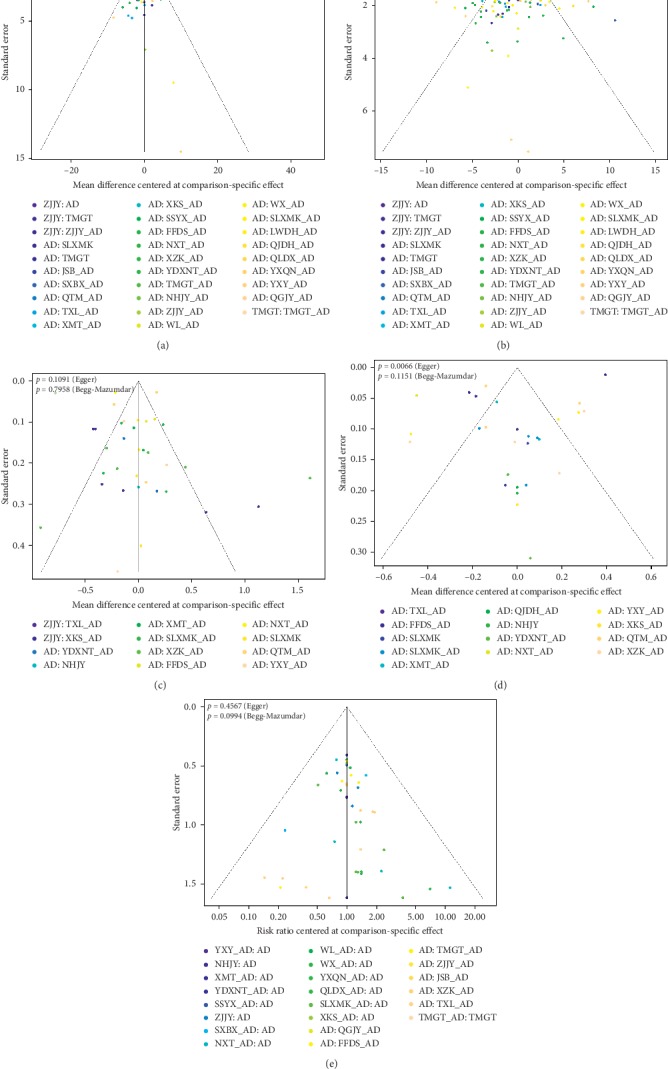
Funnel plots. (a) Systolic blood pressure. (b) Diastolic blood pressure. (c) Total cholesterol. (d) Triglyceride. (e) Adverse effects.

*(4) Publication Bias*. All the outcomes were analyzed in funnel plots with Egger's and Begg's tests to detect the small-study effects and publication bias. The tests of DBP, TC, and adverse effects showed no significant difference in symmetry while that of SBP and TG showed inconsistent results in two tests which mean publication bias might exist (SBP : Egger's *P*=0.04, Begg's *P*=0.977; TG : Egger's *P*=0.007, Begg's *P*=0.115) (Figure 6).

## 4. Discussion

### 4.1. Findings and Interpretations

In this network meta-analysis of all the TCPM for primary hypertension, we found that most TCPM combined with AD can significantly reduce both SBP and DBP. For SBP reduction, eighteen interventions had significant differences compared with AD. According to the treatment ranking probabilities, YXY + AD was superior to others, followed by LWDH + AD, NHJY, and QGJY + AD. For DBP reduction, sixteen interventions were significantly better than AD. Among them, QGJY + AD and QJDH + AD were superior to others, and they were followed by YXQN + AD. To summarize the SUCRA values results, we found that QGJY + AD and YXY + AD had the most significant reductions for both SBP and DBP. In accordance with league table for network comparisons, we found that QGJY + AD had no significant difference comparing with YXY + AD, which made QGJY + AD and YXY + AD the best options for SBP and DBP reductions.

For the secondary outcomes, we found that some TCPMs can improve the blood TC and TG levels. For the TC level, seven interventions were significantly better than AD alone. Among them, YXY + AD showed the most reductions and was followed by QTM + AD. For the TG level, three interventions were significantly better than AD. Moreover, QTM + AD, XZK + AD, and YXY + AD were superior to others. In summary, YXY + AD was the best one for both TC and TG control.

For adverse effects, we found that only two interventions had significant differences when compared with AD alone. According to the ranking probabilities, eight interventions had less risk than AD and YXY + AD had the least risk than others. For adverse events, we found that some events happened less when AD is combined with TCPM, which included headache and dizziness, facial flush, respiratory diseases, cardiovascular disease, edema, and so forth. It indicates that combinations of AD and TCPM could improve the adverse risk of AD to a certain extent. However, we also found that some TCPMs, including SLXMK and TXL, could cause increased events of the gastrointestinal tract, which focused on upper abdominal discomfort, nausea, and emesis. These results mentioned above suggest that the adverse effects of TCPM combined with AD are not certain, and evidence is inconsistent. Thus, TCPM combined with AD should be used with caution, and more high-quality RCTs are required to explore it.

The main component of YXY is Ginkgo biloba extract (GBE), which is one of the most common complementary therapies for cardiovascular diseases due to its antioxidant, anti-inflammatory, antiplatelet and vasodilator properties [45, 46]. One previous study found that anti-inflammatory activity and antihypertension effects were potentiated when combination treatment of GBE and AD was used [47]. Another study [48] suggested that the antihypertension effect of GBE was unsatisfactory and this conclusion is inconsistent with our study. The reason for that may be the discrepancy between different races of included participants. There have been some disputes on races for treatment of antihypertension and some studies suggested that different races of patients might cause inconsistent results in treatment [49–52]. Besides, that study [48] did not concentrate on dyslipidemia in hypertension patients, and this could be another reason. Similarly, one more meta-analysis study also suggested the same result above, which reported fewer outcomes in SBP and DBP but more clinical efficacy rates that may lead to a discrepancy [53]. There were a series of studies for hypertension in China, which suggested that dyslipidemia was one of risk factors of hypertension, and hyperlipidemia was associated with increased risk of hypertension [54–57]. The main effects of lowering blood lipids of GBE were sourced from flavonoids which could improve blood lipids through antioxidant effect and blood pressure reductions were achieved then [58–61]. In our study, there were some trials that reported two outcomes on blood lipids which were TC and TG. We found that YXY + AD could be effective for hypertension patients who had dyslipidemia. But more studies are needed to explore the association between lowering blood lipids and antihypertension. QGJY is composed of many kinds of Chinese herbals. although it seems to be effective for hypertension, its mechanisms have not been explained fully. More related studies are required to clarify why it works.

In summary above, TCPM combined with AD may have more efficacy than AD alone to lower SBP and DBP while lowering TC and TG at the same time. In all sorts of TCPMs, QGJY + AD and YXY + AD seem to be the best options. Especially, YXY + AD had a satisfactory efficacy for patients of hypertension with dyslipidemia. However, the adverse effects were uncertain due to the inconsistent evidence and the balance between benefits and risks is needed.

### 4.2. Strength and Limitation

There are several strengths to our study. First, all the previous studies of TCPM for hypertension used effective clinical rates that were with inconsistent criteria as the primary outcome which could cause lager between-study heterogeneity. Our study strictly used the unified criteria of SBP and DBP as primary outcomes. Second, this is the first network meta-analysis to compare each TCPM efficacy and rank them. For many types of TCPM in China, network comparison is a very useful tool to screen out ineffective ones. Finally, primary hypertension is a chronic disease whose treatment can be influenced by many baseline conditions, including different types of ADs, age, treatment duration, course of disease, and complications. Thus, we conducted metaregressions with these confounders to have a robust result.

However, the results may be influenced by some limitations. First, most included studies were in Chinese and only two in English, whose qualities were low because of no mentions of allocation concealment and blinding. Thus, internal validity of our study was low, and caution is needed for using our evidence. Second, in the analysis of blood lipids, only two indicators (TC, TG) were reported and compared, but others (low-density lipoprotein cholesterol, high-density lipoprotein cholesterol, etc.) were not. Therefore, a comprehensive analysis of blood lipids was required for more precise results. Third, some interventions had wide credible intervals due to the small amount of included studies, which implies that these results might not be a valuable reference. Besides, some studies reported a few adverse events, especially in detail, which may have selective bias. Next, long length of follow-up is needed, but most included studies only had relatively short treatment duration with median of eight weeks and did not mention follow-up. Thus, further study is required on efficacy of long-term treatment of TCPM. Finally, on publication bias detection, two outcomes (SBP and TG) had inconsistent results in Egger's and Begg's tests which may have exaggerated results.

## 5. Conclusion

In our study, QGJY + AD and YXY + AD may be the best options for hypertension. Besides, YXY + AD can improve blood lipids in patients with hypertension and more related studies on the association of blood lipids and hypertension are needed. Most TCPMs seem to be effective for hypertension but due to the quality of studies and other limitations, more high-quality RCTs are required to prove it.

## Figures and Tables

**Table 1:** League table of Systolic blood pressure (SBP) and Diastolic blood pressure (DBP).

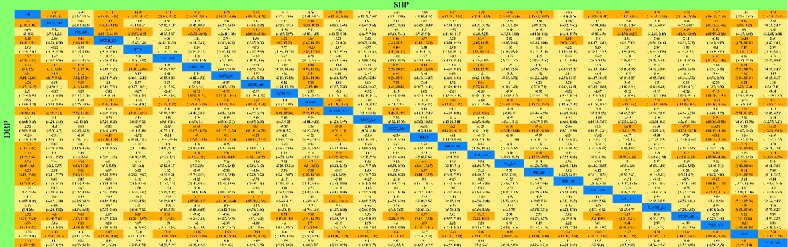

Dark brown represents statistical significance. Light brown represents no statistical significance. SBP: Systolic blood pressure. DBP: Diastolic blood pressure.

**Table 2:** The SUCRA of outcomes.

Interventions	SBP	DBP	Adjustment of DBP^*∗*^	TC	TG	AEs
QGJY + AD	0.78	0.89	0.88	—	—	0.65
YXY + AD	0.91	0.74	0.79	0.84	0.67	0.01
YXQN + AD	0.73	0.88	0.86	—	—	0.53
QLDX + AD	0.75	0.85	0.87	—	—	0.54
QJDH + AD	0.66	0.89	0.94	—	0.46	—
LWDH + AD	0.86	0.68	0.61	—	—	—
SLXMK + AD	0.63	0.85	0.85	0.48	0.41	0.55
WX + AD	0.73	0.69	0.57	—	—	0.45
WL + AD	0.68	0.69	0.63	—	—	0.45
ZJJY + AD	0.67	0.55	0.63	—	—	0.74
NHJY	0.8	0.35	0.42	0.42	0.51	0.19
TMGT + AD	0.62	0.52	0.57	—	—	0.68
YDXNT + AD	0.61	0.49	0.56	0.32	0.54	0.2
XZK + AD	0.69	0.27	0.58	0.61	0.81	0.83
NXT + AD	0.39	0.57	0.29	0.68	0.61	0.37
SSYX + AD	0.33	0.55	0.48	—	—	0.27
FFDS + AD	0.44	0.44	0.38	0.64	0.35	0.68
XKS + AD	0.35	0.52	0.49	0.31	0.69	0.59
XMT + AD	0.42	0.44	0.53	0.42	0.44	0.19
TXL + AD	0.25	0.38	0.33	0.27	0.21	0.95
QTM + AD	0.26	0.32	0.39	0.75	0.79	—
SXBX + AD	0.18	0.3	0.27	—	—	0.29
JSB + AD	0.24	0.17	0.09	—	—	0.75
TMGT	0.21	0.14	0.17	—	—	0.71
SLXMK	0.11	0.18	0.19	0.7	0.4	—
AD	0.06	0.1	0.1	0.04	0.12	0.6
ZJJY	0.13	0.02	0.03	—	—	0.27

SBP: systolic blood pressure. DBP: diastolic blood pressure. TC: otal cholesterol. TG: triglyceride. AEs: adverse effects. ^*∗*^SUCRA of DBP after adjustment for significant covariates in metaregression.

## Data Availability

The categorical and continuous variables supporting this Network Meta-Analysis are from previously reported studies and datasets, which have been cited. The processed data are available in the supplementary files (S3 Supplementary Data).

## Supplementary Materials

Supplementary Materials The categorical and continuous variables supporting this Network Meta-Analysis are from previously reported studies and datasets, which have been cited. The processed data are available in the supplementary files (S3 Supplementary Data).
