# Supplementary material for: Traditional Chinese Patent Medicine for Primary Hypertension: A Bayesian Network Meta-Analysis
Source: Evid Based Complement Alternat Med. 2020 Apr 24;2020:6701272. doi: 10.1155/2020/6701272 (PMC7196995; doi:10.1155/2020/6701272)
Supplement: Supplementary Materials — The categorical and continuous variables supporting this Network Meta-Analysis are from previously reported studies and datasets, which have been cited. The processed data are available in the supplementary files (S3 Supplementary Data). [file 6701272.f1.docx]

**S3 Supplementary Data**

**Systolic blood pressure (SBP)**

| study | Treatment | Control | Treatment Mean | Treatment SD | Treatment sample size | Control Mean | Control SD | Control sample size |
| --- | --- | --- | --- | --- | --- | --- | --- | --- |
| Cai 2004 | LWDH_AD | AD | -47 | 5.222327068 | 93 | -35 | 5.400064814 | 61 |
| Cai 2012 | YXY_AD | AD | -46.8 | 13.35252785 | 80 | -28.7 | 8.534635317 | 80 |
| Chai 2007 | FFDS_AD | AD | -22.95 | 17.51165683 | 50 | -24.975 | 14.31675155 | 50 |
| Chang 2012 | SLXMK_AD | AD | -30.1 | 9.361623791 | 51 | -17.8 | 9.614052215 | 49 |
| Chen 2006 | YXQN_AD | AD | -24 | 6.075360072 | 64 | -9.5 | 7.050531895 | 66 |
| Chen 2008 | LWDH_AD | AD | -45.75 | 10.83769463 | 49 | -35.25 | 11.16140112 | 48 |
| Chen 2011a | QTM_AD | AD | -21 | 9.539392014 | 327 | -18 | 10.58300524 | 173 |
| Chen 2011b | SXBX_AD | AD | -30.9 | 5.291502622 | 65 | -30.3 | 4.757099957 | 65 |
| Chen 2013a | QGJY_AD | AD | -39.9 | 6.536818798 | 108 | -29.8 | 7.562407024 | 108 |
| Chen 2013b | FFDS_AD | AD | -45.7 | 10.71400952 | 120 | -32.5 | 11.52085066 | 120 |
| Chen 2013c | FFDS_AD | AD | -33 | 10.58300524 | 80 | -23 | 10.14889157 | 80 |
| Chen 2013d | SXBX_AD | AD | -29 | 6.244997998 | 64 | -28 | 6 | 64 |
| Chen 2014a | XZK_AD | AD | -20.09 | 10.41350565 | 33 | -15.34 | 9.502699616 | 32 |
| Chen 2014b | TMGT_AD | AD | -34.13 | 8.245368397 | 60 | -24.81 | 11.16597958 | 60 |
| Chen 2015 | WL_AD | AD | -31.2 | 8.784645696 | 48 | -19.9 | 9.180413934 | 48 |
| Cheng 2007 | LWDH_AD | AD | -46.575 | 5.176630178 | 282 | -34.875 | 5.331334261 | 276 |
| Cheng 2011 | TXL_AD | AD | -43 | 24.0208243 | 68 | -43 | 23.89560629 | 40 |
| Cui 2013 | AD | ZJJY | -24 | 11.13552873 | 50 | -16 | 13.11487705 | 50 |
| Dai 2006 | NHJY | AD | -13 | 8.268470233 | 33 | -7.5 | 7.456455257 | 30 |
| Deng 2008 | FFDS_AD | AD | -31.066 | 10.17623098 | 30 | -28 | 10.49352605 | 30 |
| Diao 2014 | YXQN_AD | AD | -29.89 | 5.668050811 | 248 | -16.73 | 5.898143776 | 248 |
| Ding 2013 | NHJY | AD | -27.1 | 12.97420518 | 52 | -11.1 | 12.16059209 | 52 |
| Ding 2014 | TMGT | AD | -21.5 | 11.7999322 | 30 | -19.34 | 11.87980219 | 30 |
| Dong 2009 | QJDH_AD | AD | -27 | 7.003684744 | 60 | -26.84 | 11.74047273 | 60 |
| Dong 2014 | YXY_AD | AD | -19.5 | 6.408002809 | 50 | -7.5 | 7.232738071 | 50 |
| Dong 2015a | YXQN_AD | AD | -37.2 | 10.13854033 | 43 | -26.2 | 10.93800713 | 43 |
| Dong 2015b | FFDS_AD | AD | -28.4 | 10.15332458 | 43 | -28 | 10.69252075 | 43 |
| Fang 2012 | ZJJY | AD | -36.8 | 12.91084815 | 142 | -38.9 | 13.17611475 | 142 |
| Fang 2019 | YXY_AD | AD | -38 | 14.73091986 | 100 | -18 | 14.79864859 | 100 |
| Feng 2012 | JSB_AD | AD | -37.4 | 10.5399241 | 42 | -35.5 | 9.887871358 | 42 |
| Feng 2014 | YDXNT_AD | AD | -26.9 | 12.30121945 | 50 | -17.2 | 10.3696673 | 50 |
| Fu 2009a | JSB_AD | AD | -34.3 | 12.25030612 | 35 | -30.8 | 11.00363576 | 30 |
| Fu 2009b | SLXMK_AD | AD | -27 | 12.77380131 | 35 | -19 | 11.13148687 | 35 |
| Fu 2012 | FFDS_AD | AD | -30 | 10.73498952 | 32 | -25 | 12.20122945 | 32 |
| Gao 2012 | YDXNT_AD | AD | -27.3 | 14.50652612 | 57 | -27.02 | 14.33637332 | 59 |
| Gao 2013 | SLXMK_AD | AD | -26.74 | 12.06999586 | 60 | -17.07 | 13.59388465 | 60 |
| Gao 2017 | YXQN_AD | AD | -29.18 | 14.24658907 | 293 | -16.02 | 15.40403843 | 293 |
| Gong 2010 | XZK_AD | AD | -86 | 21.9317122 | 32 | -32 | 19 | 30 |
| Gu 2003 | SLXMK | AD | -23 | 13.89244399 | 45 | -24 | 12.12435565 | 40 |
| Gu 2010 | XZK_AD | AD | -9.6 | 13.62938003 | 73 | -1.3 | 15.90094337 | 62 |
| Gui 2017 | YDXNT_AD | AD | -36.61 | 13.06517891 | 101 | -20.29 | 13.9813769 | 100 |
| Guo 2016 | XZK_AD | AD | -37.66 | 8.95083795 | 60 | -33.16 | 9.17887248 | 60 |
| He 2006 | SLXMK_AD | AD | -31.1 | 11.13956911 | 60 | -24.4 | 12.53953747 | 58 |
| He 2009 | FFDS_AD | AD | -28 | 10.73498952 | 40 | -27 | 12.68581885 | 40 |
| He 2012 | ZJJY | AD | -19.3 | 12.35758876 | 60 | -11.7 | 11.60517126 | 60 |
| He 2015 | WX_AD | AD | -9.7 | 12.57417989 | 48 | -10 | 12.94179277 | 44 |
| Hong 2014 | YXY_AD | AD | -35.1 | 4.828043082 | 65 | -26.6 | 5.411099703 | 65 |
| Hou 2010 | ZJJY | AD | -36.2 | 8.613361713 | 48 | -16.6 | 6.67757441 | 42 |
| Hu 2011a | NXT_AD | AD | -20.2 | 15.42433143 | 60 | -20 | 14.95025083 | 60 |
| Hu 2011b | NXT_AD | AD | -28.3 | 9.392017888 | 60 | -23.7 | 10.6409586 | 60 |
| Hu 2012 | TXL_AD | AD | -48 | 13.11487705 | 50 | -45 | 13.45362405 | 50 |
| Hu 2016 | WL_AD | AD | -33.6 | 8.30722577 | 25 | -22.3 | 8.453993139 | 25 |
| Huang 2009 | FFDS_AD | AD | -26 | 10.44557323 | 33 | -24 | 11.20892502 | 33 |
| Huang 2012 | AD | ZJJY | -25.3 | 5.802585631 | 40 | -26.1 | 7.05336799 | 35 |
| Huang 2016 | QJDH_AD | AD | -40.9 | 8.80170438 | 50 | -30.5 | 10.77636302 | 50 |
| Huang 2018 | QLDX_AD | AD | -22.69 | 8.291085574 | 39 | -14.12 | 7.628623729 | 39 |
| Ji 2011 | YXQN_AD | AD | -36.12 | 8.663965605 | 80 | -29.46 | 8.004142677 | 80 |
| Jiang 2011 | SLXMK_AD | AD | -34.12 | 9.340380078 | 52 | -24.22 | 8.962661435 | 50 |
| Jin 2001 | SLXMK | AD | -18.75 | 17.03672504 | 60 | -21.75 | 14.63941597 | 60 |
| Kang 2017 | YXQN_AD | AD | -33.81 | 5.625539974 | 30 | -23.01 | 6.263449529 | 30 |
| Ke 2007 | XZK_AD | AD | -10 | 6 | 30 | -2 | 6.557438524 | 30 |
| Lan 2015 | SLXMK_AD | AD | -29.15 | 7.605576901 | 25 | -22.92 | 7.35920512 | 25 |
| Li 2003 | TXL_AD | AD | -18.1 | 2.1 | 44 | -10.8 | 1.3 | 42 |
| Li 2006a | YXQN_AD | AD | -12 | 6.634003316 | 41 | -8 | 6.030754513 | 40 |
| Li 2006b | YXQN_AD | AD | -42.35 | 11.522452 | 40 | -38.42 | 11.52001302 | 40 |
| Li 2008 | XKS_AD | AD | -22.07 | 4.943156886 | 36 | -19.24 | 4.903427373 | 36 |
| Li 2009 | SXBX_AD | AD | -22.1 | 4.887739764 | 36 | -19.2 | 4.877499359 | 36 |
| Li 2010 | SSYX_AD | AD | -30 | 15.76959099 | 36 | -28 | 17.30086703 | 32 |
| Li 2011a | SLXMK_AD | AD | -21.5 | 11.34592438 | 60 | -9.4 | 11.5112988 | 60 |
| Li 2011b | SLXMK_AD | AD | -39.04 | 13.87722234 | 39 | -29.8 | 15.00138994 | 39 |
| Li 2011c | FFDS_AD | AD | -30 | 10.81665383 | 29 | -15 | 13.22875656 | 27 |
| Li 2014 | ZJJY_AD | AD | -53.1 | 29.41581207 | 45 | -42.9 | 37.23076685 | 45 |
| Li 2016 | SLXMK_AD | AD | -42 | 8.185352772 | 118 | -31 | 8.888194417 | 118 |
| Li 2017 | TXL_AD | AD | -17.93 | 10.77502668 | 36 | -10.17 | 10.47159491 | 36 |
| Li 2018 | NXT_AD | AD | -33.5 | 14.64274564 | 100 | -23.8 | 14.66867411 | 100 |
| Liu 2009 | YXQN_AD | AD | -31.1 | 11.13956911 | 60 | -24.4 | 12.53953747 | 58 |
| Liu 2012 | QTM_AD | AD | -24.7 | 14.31328055 | 50 | -22.2 | 15.13175469 | 50 |
| Liu 2013a | XZK_AD | AD | -31.77 | 6.446184918 | 38 | -23.34 | 5.873567911 | 39 |
| Liu 2013b | QLDX_AD | AD | -24.6 | 10.17251198 | 48 | -14.3 | 10.9672239 | 48 |
| Liu 2014 | TMGT_AD | AD | -27.7 | 8.515867542 | 63 | -17.3 | 8.549268975 | 64 |
| Liu 2015a | QGJY_AD | AD | -38.9 | 15.06220435 | 89 | -18.5 | 14.38158545 | 89 |
| Liu 2015b | XMT_AD | AD | -43.7 | 20.26894176 | 50 | -38 | 17.90837793 | 50 |
| Liu 2016a | YXQN_AD | AD | -35.4 | 9.403755899 | 45 | -25.725 | 10.05 | 45 |
| Liu 2016b | ZJJY | ZJJY_AD | -23.6 | 15.12249979 | 33 | -23.7 | 12.82926342 | 27 |
| Liu 2017 | NXT_AD | AD | -19.7 | 9.180038126 | 60 | -11.76 | 8.886906098 | 60 |
| Liu 2018 | FFDS_AD | AD | -31.8 | 7.794870108 | 40 | -30.6 | 7.644605941 | 41 |
| Luo 2013 | XMT_AD | AD | -14.4 | 10.81058278 | 58 | -8.81 | 11.91613612 | 58 |
| Man 2014 | SLXMK_AD | AD | -38.3 | 9.656603958 | 45 | -36.5 | 10.37641557 | 40 |
| Mao 2017 | NXT_AD | AD | -33.98 | 10.78710805 | 81 | -20.22 | 11.0717117 | 80 |
| Meng 2014 | ZJJY_AD | AD | -12.4 | 9.914131329 | 32 | -7.7 | 8.506468127 | 28 |
| Mo 2009 | TXL_AD | AD | -27 | 10.53565375 | 120 | -29 | 9.539392014 | 120 |
| Niu 2018 | QJDH_AD | AD | -28.23 | 4.538678222 | 31 | -13.75 | 4.763013752 | 31 |
| OuYang 2002 | QJDH_AD | AD | -33.75 | 7.154544011 | 34 | -29.25 | 11.09898644 | 34 |
| Peng 2016 | NXT_AD | AD | -21.24 | 9.324489262 | 35 | -16.21 | 9.595316566 | 40 |
| Peng 2017 | SLXMK_AD | AD | -18.5 | 2.70700942 | 75 | -7.99 | 2.465197761 | 75 |
| Peng 2018 | WX_AD | AD | -29.1 | 13.65906293 | 60 | -13.2 | 13.91725548 | 60 |
| Qin 2003 | YXQN_AD | AD | -21.975 | 4.378998173 | 50 | -9 | 5.025 | 50 |
| Qiu 2015 | WX_AD | AD | -38.61 | 17.21946283 | 66 | -32.8 | 18.5959216 | 63 |
| Shen 1998 | ZJJY | AD | -18.75 | 13.5 | 27 | -20.25 | 14.0662495 | 25 |
| Shen 2007 | NXT_AD | AD | -24.5 | 16.55483011 | 30 | -25 | 14.12871898 | 30 |
| Sheng 2013 | ZJJY_AD | AD | -28.7 | 7.528632014 | 78 | -22.53 | 7.890304177 | 72 |
| Shi 2014 | YXY_AD | AD | -23.1 | 13.91492364 | 30 | -8.48 | 13.24013217 | 30 |
| Shi 2015 | YXQN_AD | AD | -32.9 | 7.52861209 | 199 | -19.8 | 9.282779756 | 199 |
| Shi 2016 | YXQN_AD | AD | -29.73 | 8.984525586 | 42 | -20.64 | 9.925034005 | 41 |
| Su 2014 | YXQN_AD | AD | -29.17 | 14.26695132 | 50 | -22 | 15.41711062 | 50 |
| Sun 2015 | YXY_AD | AD | -20.2 | 7.514652354 | 35 | -10.3 | 7.518643495 | 35 |
| Sun 2017a | SLXMK_AD | AD | -24.4 | 7.3430239 | 35 | -20 | 6.550572494 | 35 |
| Sun 2017b | SLXMK | AD | -7.3 | 14.78242199 | 60 | -8.5 | 15.69044295 | 30 |
| Tan 2018 | QTM_AD | AD | -17.8 | 9.383197749 | 97 | -13.78 | 9.014837769 | 97 |
| Tao 2013 | SXBX_AD | AD | -27.3 | 10.55130324 | 56 | -21.8 | 12.50479908 | 54 |
| Wang 1999 | SLXMK | AD | -22 | 14.73091986 | 36 | -24 | 12.12435565 | 30 |
| Wang 2004 | SLXMK | AD | -23 | 13.89244399 | 45 | -24 | 12.12435565 | 40 |
| Wang 2005 | SLXMK_AD | AD | -36.75 | 12.64977075 | 20 | -29.15 | 9.941005985 | 20 |
| Wang 2006 | SLXMK | AD | -20 | 13.85640646 | 46 | -22 | 14.73091986 | 39 |
| Wang 2007 | XZK_AD | AD | -21.7 | 14.32724677 | 30 | -16.8 | 13.95600229 | 30 |
| Wang 2010 | NXT_AD | AD | -26 | 10.44557323 | 35 | -24 | 11.20892502 | 35 |
| Wang 2011a | SLXMK_AD | AD | -35.3 | 9.638983349 | 70 | -29.7 | 10.77636302 | 68 |
| Wang 2011b | TXL_AD | AD | -29.2 | 9.801530493 | 97 | -18.9 | 8.845337755 | 97 |
| Wang 2012 | YDXNT_AD | AD | -13.24 | 11.77769502 | 40 | -8.17 | 10.61025447 | 37 |
| Wang 2014a | SLXMK_AD | AD | -39.26 | 7.092369139 | 60 | -29.47 | 7.577618359 | 60 |
| Wang 2014b | YXY_AD | AD | -17.15 | 9.286700167 | 70 | -7.65 | 9.789080651 | 70 |
| Wang 2014c | ZJJY_AD | AD | -23.6 | 15.12249979 | 34 | -13.7 | 12.82926342 | 34 |
| Wang 2015 | YXY_AD | AD | -25 | 23.2594067 | 48 | -21 | 23.06512519 | 48 |
| Wang 2017 | QGJY_AD | AD | -44.9 | 6.593936609 | 43 | -31.3 | 6.255397669 | 43 |
| Wang 2018 | SLXMK | AD | -10.8 | 9.41 | 137 | -10.01 | 9.7 | 139 |
| Wei 2009 | TXL_AD | AD | -32 | 21.63330765 | 43 | -33 | 22.5166605 | 50 |
| Wei 2014 | TMGT_AD | TMGT | -41.6 | 6.57951366 | 64 | -32.6 | 7.274613392 | 64 |
| Weng 2011 | SLXMK_AD | AD | -30.98 | 7.719410599 | 45 | -22.07 | 6.216840033 | 45 |
| Weng 2015 | SLXMK_AD | AD | -32.9 | 10.75546373 | 80 | -25.1 | 11.58576713 | 80 |
| Weng 2016 | SLXMK_AD | AD | -49.94 | 11.21484284 | 43 | -42.16 | 11.81531633 | 43 |
| Wu 2002 | SLXMK | AD | -26.25 | 13.90476537 | 45 | -8.55 | 6.972580225 | 35 |
| Wu 2005 | AD | ZJJY | -36.1 | 8.055432949 | 108 | -24 | 6.495382976 | 108 |
| Wu 2006 | XZK_AD | AD | -18 | 13.89244399 | 60 | -12 | 15.13274595 | 60 |
| Wu 2009 | XZK_AD | AD | -12 | 14.17744688 | 27 | -9 | 15.39480432 | 28 |
| Wu 2016 | YXQN_AD | AD | -35.9 | 74.2317459 | 29 | -16.5 | 24.23076763 | 29 |
| Xie 2012 | WL_AD | AD | -26.69 | 11.48056183 | 30 | -15.1 | 11.53754307 | 30 |
| Xie 2014 | YXQN_AD | AD | -26.3 | 11.72859753 | 45 | -24.7 | 13.32929105 | 45 |
| Xie 2015 | XZK_AD | AD | -31.3 | 11.74265728 | 36 | -16.3 | 13.08472392 | 36 |
| Xie 2016 | SLXMK_AD | AD | -15.44 | 5.179073276 | 120 | -8.62 | 2.935762252 | 120 |
| Xie 2017 | SLXMK_AD | AD | -22.3 | 12.74244874 | 44 | -13 | 14.19048977 | 46 |
| Xin 2013 | TXL_AD | AD | -1.07 | 14.28622063 | 30 | 0.84 | 12.30004878 | 30 |
| Xiong 2012 | XZK_AD | AD | -56.8 | 8.685044617 | 60 | -45.1 | 8.328865469 | 60 |
| Xiong 2018 | QLDX_AD | AD | -25 | 10.14889157 | 63 | -11 | 11.13552873 | 63 |
| Xu 2007 | TXL_AD | AD | -22.07 | 4.943156886 | 36 | -21.32 | 4.446616691 | 36 |
| Xu 2010 | TMGT | ZJJY | -30 | 17.34935157 | 30 | -24 | 17.77638883 | 30 |
| Xu 2012 | XKS_AD | AD | -36.5 | 7.968061245 | 83 | -34.3 | 7.562407024 | 84 |
| Xu 2013 | XZK_AD | AD | -20.9 | 11.74606317 | 30 | -18 | 13.51258672 | 30 |
| Xu 2015a | NXT_AD | AD | -14 | 7.408103671 | 90 | -6.8 | 7.418220811 | 90 |
| Xu 2015b | SLXMK_AD | AD | -35.3 | 10.25036585 | 40 | -26.4 | 10.94212045 | 40 |
| Yan 2010a | JSB_AD | AD | -14.27 | 10.69050513 | 65 | -14.47 | 11.03498527 | 50 |
| Yan 2010b | JSB_AD | AD | -38.6 | 11.79364236 | 46 | -34.6 | 11.6090482 | 46 |
| Yan 2013 | YXQN_AD | AD | -31.1 | 10.05385498 | 39 | -22.5 | 11.26809656 | 39 |
| Yan 2015 | XMT_AD | AD | -19.02 | 11.34620641 | 60 | -11.57 | 10.65022535 | 60 |
| Yan 2017 | QGJY_AD | AD | -39.9 | 6.437390776 | 100 | -29.8 | 7.462573283 | 100 |
| Yang 2006 | FFDS_AD | AD | -47 | 5.222327068 | 286 | -35 | 5.400064814 | 275 |
| Yang 2015 | TXL_AD | AD | -33.71 | 4.571640843 | 34 | -33.14 | 5.190645432 | 48 |
| Ye 2006 | XZK_AD | AD | -18 | 13.89244399 | 51 | -12 | 15.13274595 | 49 |
| Ye 2009 | XZK_AD | AD | -12 | 14.17744688 | 28 | -9 | 15.39480432 | 27 |
| You 2014 | FFDS_AD | AD | -33 | 10.14889157 | 34 | -22 | 14.93318452 | 34 |
| Yu 2005 | TXL_AD | AD | -29.8 | 9.704122835 | 27 | -17.8 | 8.825531145 | 25 |
| Yuan 2008 | SXBX_AD | AD | -33.61 | 12.0719841 | 40 | -32.1 | 12.17139269 | 40 |
| Yuan 2013 | NXT_AD | AD | -27 | 10.53565375 | 100 | -29 | 9.848857802 | 100 |
| Zhang 2006a | LWDH_AD | AD | -47 | 5.212331532 | 70 | -32 | 5.295573623 | 66 |
| Zhang 2006b | LWDH_AD | AD | -43.5 | 35.15412494 | 34 | -24 | 42.59181259 | 34 |
| Zhang 2011 | FFDS_AD | AD | -30.2 | 15.4181711 | 40 | -25.7 | 15.34242484 | 40 |
| Zhang 2013 | JSB_AD | AD | -30.39 | 13.77173555 | 53 | -24.48 | 12.75561445 | 53 |
| Zhang 2016a | QGJY_AD | AD | -10.9 | 4.667901027 | 47 | -8.6 | 4.260880191 | 44 |
| Zhang 2016b | TXL_AD | AD | -35.9 | 15.25417976 | 52 | -36.6 | 15.97717121 | 50 |
| Zhang 2016c | WL_AD | AD | -14.76 | 4.5 | 76 | -11.45 | 3.06 | 78 |
| Zhang 2016d | SXBX_AD | AD | -30.7 | 15.56534613 | 82 | -29.9 | 16.86534909 | 82 |
| Zhao 2010 | TXL_AD | AD | -32.94 | 10.06126732 | 45 | -32.99 | 9.527575767 | 45 |
| Zhao 2012 | QGJY_AD | AD | -41.9 | 7.238093672 | 40 | -35.6 | 6.781592733 | 40 |
| Zhao 2013 | XKS_AD | AD | -36.9 | 8.122191822 | 39 | -35.8 | 9.924212815 | 39 |
| Zhao 2016 | SSYX_AD | AD | -44.625 | 12.90632113 | 31 | -30.225 | 11.76113834 | 31 |
| Zhao 2018a | SLXMK_AD | AD | -24.46 | 12.0324852 | 47 | -16.62 | 12.29411241 | 47 |
| Zhao 2018b | XKS_AD | AD | -42.52 | 8.590628615 | 43 | -29.64 | 8.88554444 | 43 |
| Zheng 2016b | WX_AD | AD | -31.1 | 16.32554134 | 80 | -14.51 | 14.21465441 | 80 |
| Zheng 2017 | QLDX_AD | AD | -23.04 | 2.455137471 | 50 | -15.66 | 2.81510213 | 50 |
| Zhong 2017 | TMGT_AD | AD | -39.74 | 10.28145904 | 92 | -30.98 | 10.34835253 | 92 |
| Zhou 2003 | LWDH_AD | AD | -38 | 8.660254038 | 50 | -32 | 9.848857802 | 50 |
| Zhou 2006 | NHJY | AD | -38.4 | 20.86725904 | 160 | -26.7 | 21.08180495 | 120 |
| Zhou 2013 | TMGT_AD | AD | -25.8 | 10.84758038 | 48 | -20.2 | 11.18793994 | 49 |
| Zhou 2015 | ZJJY_AD | AD | -22 | 17.9702532 | 100 | -2.2 | 18.48864516 | 100 |
| Zhou 2018 | SSYX_AD | AD | -28.7 | 13.43093444 | 59 | -32.1 | 13.21816931 | 59 |
| Zhu 2010 | TMGT_AD | AD | -35.5 | 5.479771893 | 30 | -31.64 | 5.81758541 | 30 |
| Zhu 2012 | QJDH_AD | AD | -23.04 | 14.54950515 | 46 | -8.6 | 15.06299771 | 46 |
| Zhu 2013 | NXT_AD | AD | -48.32 | 15.59025657 | 40 | -38.37 | 14.93930721 | 40 |
| Zhu 2015a | XMT_AD | AD | -7 | 2.645751311 | 100 | -3 | 5.567764363 | 100 |
| Zhu 2015b | ZJJY_AD | AD | -14.15 | 8.415895674 | 110 | -4.07 | 8.709173325 | 108 |
| Zhu 2017 | SLXMK_AD | AD | -35.3 | 10.22203502 | 80 | -27.2 | 11.50521621 | 80 |
| Zhu 2018 | SLXMK | AD | -2.6 | 10.46135746 | 60 | -7.5 | 16.45083585 | 30 |
| Zhuang 2018 | TXL_AD | AD | -38.38 | 14.81967948 | 44 | -34.74 | 14.76724754 | 44 |

**Diastolic blood pressure (DBP)**

| study | Treatment | Control | Treatment Mean | Treatment SD | Treatment Sample Size | Control Mean | Control SD | Control Sample Size |
| --- | --- | --- | --- | --- | --- | --- | --- | --- |
| Cai 2004 | LWDH_AD | AD | -26 | 5.22045975 | 93 | -18 | 5.366292948 | 61 |
| Cai 2012 | YXY_AD | AD | -29.6 | 13.49703671 | 80 | -15.2 | 9.406380813 | 80 |
| Chai 2007 | FFDS_AD | AD | -13.95 | 8.062761624 | 50 | -15 | 7.733732928 | 50 |
| Chang 2012 | SLXMK_AD | AD | -20.8 | 5.011985634 | 51 | -8.2 | 4.912229636 | 49 |
| Chen 2006 | YXQN_AD | AD | -19 | 9.013878189 | 64 | -13 | 7.825599019 | 66 |
| Chen 2008 | LWDH_AD | AD | -26.25 | 11.02576528 | 49 | -20.25 | 11.58913823 | 48 |
| Chen 2011a | QTM_AD | AD | -15 | 8.888194417 | 327 | -13 | 7.810249676 | 173 |
| Chen 2011b | SXBX_AD | AD | -18.4 | 5.692978131 | 65 | -17.7 | 4.687216658 | 65 |
| Chen 2013a | QGJY_AD | AD | -23.2 | 8.283115356 | 108 | -10.5 | 7.118988692 | 108 |
| Chen 2013b | FFDS_AD | AD | -19.7 | 4.622769733 | 120 | -9.8 | 5.047771786 | 120 |
| Chen 2013c | FFDS_AD | AD | -23 | 7.211102551 | 80 | -18 | 6.244997998 | 80 |
| Chen 2013d | SXBX_AD | AD | -19 | 4.582575695 | 64 | -18 | 4 | 64 |
| Chen 2014a | XZK_AD | AD | -11.75 | 7.937524803 | 33 | -11.65 | 7.337894793 | 32 |
| Chen 2014b | TMGT_AD | AD | -8.58 | 8.114450074 | 60 | -7.69 | 5.573257575 | 60 |
| Chen 2015 | WL_AD | AD | -19.7 | 8.80170438 | 48 | -11 | 8.960468738 | 48 |
| Cheng 2007 | LWDH_AD | AD | -23.55 | 5.137910568 | 282 | -17.925 | 5.331334261 | 276 |
| Cheng 2011 | TXL_AD | AD | -22 | 8.544003745 | 68 | -21 | 7.549834435 | 40 |
| Cui 2013 | AD | ZJJY | -18 | 6.244997998 | 50 | -7 | 5.567764363 | 50 |
| Dai 2006 | NHJY | AD | -13 | 8.074298731 | 33 | -9.5 | 6.626720531 | 30 |
| Deng 2008 | FFDS_AD | AD | -17.3 | 6.193104149 | 30 | -15.833 | 6.660469428 | 30 |
| Diao 2014 | YXQN_AD | AD | -25.89 | 4.478582365 | 248 | -14.73 | 5.525567844 | 248 |
| Ding 2013 | NHJY | AD | -12.4 | 10.50761629 | 52 | -8.4 | 10.40144221 | 52 |
| Ding 2014 | TMGT | AD | -11.84 | 6.930216447 | 30 | -10.34 | 6.961925021 | 30 |
| Dong 2009 | QJDH_AD | AD | -17.12 | 6.289928457 | 60 | -10.49 | 3.915469321 | 60 |
| Dong 2014 | YXY_AD | AD | -12 | 6 | 50 | -6 | 49.77198007 | 50 |
| Dong 2015a | YXQN_AD | AD | -24.1 | 9.180413934 | 43 | -12.8 | 9.296773634 | 43 |
| Dong 2015b | FFDS_AD | AD | -8 | 7.922120928 | 43 | -6.5 | 8.412490713 | 43 |
| Fang 2012 | ZJJY | AD | -19.1 | 8.272242743 | 142 | -22.5 | 9.61925153 | 142 |
| Fang 2019 | YXY_AD | AD | -19 | 8.185352772 | 100 | -11 | 8.544003745 | 100 |
| Feng 2012 | JSB_AD | AD | -15 | 4.613025038 | 42 | -13.7 | 4.194043395 | 42 |
| Feng 2014 | YDXNT_AD | AD | -19.4 | 8.166394553 | 50 | -11.1 | 6.548282217 | 50 |
| Fu 2009a | JSB_AD | AD | -18.8 | 8.926925563 | 35 | -21.6 | 8.746999486 | 30 |
| Fu 2009b | SLXMK_AD | AD | -11 | 10.02646498 | 35 | -5 | 10.22350234 | 35 |
| Fu 2012 | FFDS_AD | AD | -17.5 | 8.845903006 | 32 | -15 | 8.674675786 | 32 |
| Gao 2012 | YDXNT_AD | AD | -13.8 | 8.183758305 | 57 | -12.36 | 8.063603413 | 59 |
| Gao 2013 | SLXMK_AD | AD | -12.48 | 11.00325861 | 60 | -5.15 | 10.83528034 | 60 |
| Gao 2017 | YXQN_AD | AD | -19.68 | 7.638723715 | 293 | -11.65 | 7.898379581 | 293 |
| Gong 2010 | XZK_AD | AD | -27 | 12.28820573 | 32 | -20 | 13.22875656 | 30 |
| Gu 2003 | SLXMK | AD | -20 | 8.717797887 | 45 | -20 | 7.937253933 | 40 |
| Gu 2010 | XZK_AD | AD | -2 | 7.531932023 | 73 | -1.3 | 7.95424415 | 62 |
| Gui 2017 | YDXNT_AD | AD | -18.42 | 7.422284015 | 101 | -12.3 | 7.810326498 | 100 |
| Guo 2016 | XZK_AD | AD | -18.88 | 7.063080065 | 60 | -14.73 | 7.374435572 | 60 |
| He 2006 | SLXMK_AD | AD | -28.4 | 4.757099957 | 60 | -11.8 | 7.308214556 | 58 |
| He 2009 | FFDS_AD | AD | -19 | 8.261355821 | 40 | -14 | 8.674675786 | 40 |
| He 2012 | ZJJY | AD | -13.8 | 6.502307283 | 60 | -13.1 | 6.121274377 | 60 |
| He 2015 | WX_AD | AD | -7.9 | 9.924212815 | 48 | -8.5 | 10.1 | 44 |
| Hong 2014 | YXY_AD | AD | -18.1 | 5.556077753 | 65 | -15.5 | 4.557411546 | 65 |
| Hou 2010 | ZJJY | AD | -12.7 | 5.922837158 | 48 | -10.2 | 4.132795664 | 42 |
| Hu 2011a | NXT_AD | AD | -19.5 | 9.263368718 | 60 | -19.9 | 9.001666512 | 60 |
| Hu 2011b | NXT_AD | AD | -22.6 | 7.171471258 | 60 | -15.6 | 5.211525688 | 60 |
| Hu 2012 | TXL_AD | AD | -23 | 7 | 50 | -21 | 6.557438524 | 50 |
| Hu 2016 | WL_AD | AD | -24.4 | 5.524490927 | 25 | -14.5 | 5.411099703 | 25 |
| Huang 2009 | FFDS_AD | AD | -6 | 7.549834435 | 33 | -7 | 8.544003745 | 33 |
| Huang 2012 | AD | ZJJY | -16.8 | 7.35119038 | 40 | -17.5 | 7.269112738 | 35 |
| Huang 2016 | QJDH_AD | AD | -19.6 | 5.25642464 | 50 | -13.9 | 6.509224224 | 50 |
| Huang 2018 | QLDX_AD | AD | -10.02 | 4.775196331 | 39 | -6 | 4.812525325 | 39 |
| Ji 2011 | YXQN_AD | AD | -13.93 | 5.627406152 | 80 | -11.66 | 6.317412445 | 80 |
| Jiang 2011 | SLXMK_AD | AD | -30.5 | 5.568734147 | 52 | -21.39 | 5.587477069 | 50 |
| Jin 2001 | SLXMK | AD | -7.5 | 7.611668674 | 60 | -3 | 6.873863542 | 60 |
| Kang 2017 | YXQN_AD | AD | -29.29 | 5.510970876 | 30 | -17.91 | 6.68590308 | 30 |
| Ke 2007 | XZK_AD | AD | -5 | 5 | 30 | -2 | 4.582575695 | 30 |
| Lan 2015 | SLXMK_AD | AD | -20.19 | 4.951454332 | 25 | -14.08 | 5.532440691 | 25 |
| Li 2003 | TXL_AD | AD | -10.6 | 1.4 | 44 | -5.7 | 0.6 | 42 |
| Li 2006a | YXQN_AD | AD | -15 | 4.660472079 | 41 | -15 | 3.958535083 | 40 |
| Li 2006b | YXQN_AD | AD | -3.63 | 8.498429267 | 40 | -4.24 | 8.297451416 | 40 |
| Li 2008 | XKS_AD | AD | -12.73 | 4.081801073 | 36 | -12.03 | 4.232457915 | 36 |
| Li 2009 | SXBX_AD | AD | -12.7 | 4.026164428 | 36 | -11.7 | 4.750789408 | 36 |
| Li 2010 | SSYX_AD | AD | -7 | 9.05151921 | 36 | -8 | 8.277680835 | 32 |
| Li 2011a | SLXMK_AD | AD | -17.8 | 7.158212067 | 60 | -10.5 | 7.377669009 | 60 |
| Li 2011b | SLXMK_AD | AD | -15.7 | 7.768674276 | 39 | -4.36 | 8.659514998 | 39 |
| Li 2011c | FFDS_AD | AD | -4 | 6 | 29 | -1 | 6.244997998 | 27 |
| Li 2014 | ZJJY_AD | AD | -22.2 | 21.21108201 | 45 | -19.8 | 13.08472392 | 45 |
| Li 2016 | SLXMK_AD | AD | -16 | 7 | 118 | -12 | 3.605551275 | 118 |
| Li 2017 | TXL_AD | AD | -11.12 | 8.379874701 | 36 | -5.55 | 8.446306885 | 36 |
| Li 2018 | NXT_AD | AD | -18.6 | 4.45421149 | 100 | -13 | 5.144900388 | 100 |
| Liu 2009 | YXQN_AD | AD | -28.4 | 4.757099957 | 60 | -11.8 | 7.308214556 | 58 |
| Liu 2012 | QTM_AD | AD | -17.7 | 9.10604195 | 50 | -17.4 | 9.501578816 | 50 |
| Liu 2013a | XZK_AD | AD | -1.43 | 4.400545421 | 38 | -8.82 | 5.997607856 | 39 |
| Liu 2013b | QLDX_AD | AD | -24.4 | 6.67757441 | 48 | -15.7 | 5.765414122 | 48 |
| Liu 2014 | TMGT_AD | AD | -10.4 | 6.062177826 | 63 | -4.5 | 6.302380503 | 64 |
| Liu 2015a | QGJY_AD | AD | -24.5 | 9.659710141 | 89 | -12.2 | 10.1 | 89 |
| Liu 2015b | XMT_AD | AD | -14.6 | 3.143246729 | 50 | -11.2 | 3.579106034 | 50 |
| Liu 2016a | YXQN_AD | AD | -24 | 8.421067331 | 45 | -15.45 | 8.938225495 | 45 |
| Liu 2016b | ZJJY | ZJJY_AD | -25.6 | 4.911211663 | 33 | -30.3 | 4.956813493 | 27 |
| Liu 2017 | NXT_AD | AD | -27.11 | 7.851872388 | 60 | -14.32 | 8.39822005 | 60 |
| Liu 2018 | FFDS_AD | AD | -16.6 | 3.67151195 | 40 | -13.9 | 4.357751714 | 41 |
| Luo 2013 | XMT_AD | AD | -11.87 | 6.124802038 | 58 | -8.77 | 6.815929871 | 58 |
| Man 2014 | SLXMK_AD | AD | -15.9 | 6.577993615 | 45 | -10.8 | 7.029224708 | 40 |
| Mao 2017 | NXT_AD | AD | -24.98 | 4.372424956 | 81 | -14.44 | 4.552329074 | 80 |
| Meng 2014 | ZJJY_AD | AD | -9.4 | 6.322183167 | 32 | -6.7 | 7.907591289 | 28 |
| Mo 2009 | TXL_AD | AD | -17 | 7 | 120 | -19 | 7 | 120 |
| Niu 2018 | QJDH_AD | AD | -23.39 | 4.094105519 | 31 | -12.05 | 4.151301001 | 31 |
| OuYang 2002 | QJDH_AD | AD | -12.75 | 8.649421946 | 34 | -6.75 | 8.11249037 | 34 |
| Peng 2016 | NXT_AD | AD | -8.33 | 7.156793975 | 35 | -3.27 | 7.220810204 | 40 |
| Peng 2017 | SLXMK_AD | AD | -16.9 | 2.543835687 | 75 | -11.09 | 1.830819489 | 75 |
| Peng 2018 | WX_AD | AD | -18.5 | 11.33181362 | 60 | -7.7 | 11.86465339 | 60 |
| Qin 2003 | YXQN_AD | AD | -12.75 | 3.442655225 | 50 | -6 | 2.650589557 | 50 |
| Qiu 2015 | WX_AD | AD | -28.17 | 10.39116933 | 66 | -24.37 | 10.26582193 | 63 |
| Shen 1998 | ZJJY | AD | -7.5 | 8.25 | 27 | -8.25 | 8.649421946 | 25 |
| Shen 2007 | NXT_AD | AD | -7.6 | 12.13593425 | 30 | -6 | 14.18072988 | 30 |
| Sheng 2013 | ZJJY_AD | AD | -13.95 | 5.931433216 | 78 | -8.44 | 6.455168472 | 72 |
| Shi 2014 | YXY_AD | AD | -17.96 | 7.074878091 | 30 | -9.83 | 6.682581836 | 30 |
| Shi 2015 | YXQN_AD | AD | -31.8 | 6.337980751 | 199 | -15.7 | 6.425729531 | 199 |
| Shi 2016 | YXQN_AD | AD | -34.19 | 5.287844551 | 42 | -18.52 | 5.808037534 | 41 |
| Su 2014 | YXQN_AD | AD | -15.68 | 7.632555273 | 50 | -11.62 | 7.714538223 | 50 |
| Sun 2015 | YXY_AD | AD | -13.1 | 5.910160742 | 35 | -7.2 | 5.85918083 | 35 |
| Sun 2017a | SLXMK_AD | AD | -21.8 | 5.766281297 | 35 | -15.8 | 6.337980751 | 35 |
| Sun 2017b | SLXMK | AD | -4.2 | 9.895958771 | 60 | -4.9 | 10.57875229 | 30 |
| Tan 2018 | QTM_AD | AD | -16.28 | 8.312424436 | 97 | -11.69 | 9.448576612 | 97 |
| Tao 2013 | SXBX_AD | AD | -19.4 | 12.80117182 | 56 | -6.5 | 14.07160261 | 54 |
| Wang 1999 | SLXMK | AD | -19 | 9.539392014 | 36 | -19 | 7.211102551 | 30 |
| Wang 2004 | SLXMK | AD | -20 | 8.717797887 | 45 | -20 | 7.937253933 | 40 |
| Wang 2005 | SLXMK_AD | AD | -17.75 | 12.52321045 | 20 | -10.9 | 12.23046606 | 20 |
| Wang 2006 | SLXMK | AD | -7 | 7.549834435 | 46 | -9 | 7.810249676 | 39 |
| Wang 2007 | XZK_AD | AD | -14.9 | 9.462029381 | 30 | -10.1 | 9.021640649 | 30 |
| Wang 2010 | NXT_AD | AD | -6 | 7.549834435 | 35 | -6 | 8.38808679 | 35 |
| Wang 2011a | SLXMK_AD | AD | -22.7 | 7.434379598 | 70 | -13.3 | 7.95424415 | 68 |
| Wang 2011b | TXL_AD | AD | -19.4 | 5.499090834 | 97 | -8.6 | 5.150728104 | 97 |
| Wang 2012 | YDXNT_AD | AD | -1.51 | 12.80431177 | 40 | -1.99 | 10.65380683 | 37 |
| Wang 2014a | SLXMK_AD | AD | -23.77 | 5.087012876 | 60 | -16.83 | 4.481774202 | 60 |
| Wang 2014b | YXY_AD | AD | -12.57 | 5.472101973 | 70 | -5.38 | 6.0281921 | 70 |
| Wang 2014c | ZJJY_AD | AD | -25.6 | 4.911211663 | 34 | -19.3 | 4.956813493 | 34 |
| Wang 2015 | YXY_AD | AD | -14 | 11.53256259 | 48 | -13 | 12 | 48 |
| Wang 2017 | QGJY_AD | AD | -18.8 | 7.250517223 | 43 | -10.9 | 8.201829064 | 43 |
| Wang 2018 | SLXMK | AD | -8.36 | 7.71 | 137 | -8.39 | 8.41 | 139 |
| Wei 2009 | TXL_AD | AD | -20 | 7.689603371 | 43 | -20 | 7.391210997 | 50 |
| Wei 2014 | TMGT_AD | TMGT | -19.2 | 6.317436189 | 64 | -11.5 | 6.009991681 | 64 |
| Weng 2011 | SLXMK_AD | AD | -10.36 | 7.102175723 | 45 | -9.86 | 5.705006573 | 45 |
| Weng 2015 | SLXMK_AD | AD | -24.9 | 5.502726597 | 80 | -13.4 | 6.150609726 | 80 |
| Weng 2016 | SLXMK_AD | AD | -28.17 | 5.953629145 | 43 | -20.84 | 5.933734069 | 43 |
| Wu 2002 | SLXMK | AD | -13.2 | 6.063878709 | 45 | -5.7 | 5.913596621 | 35 |
| Wu 2005 | AD | ZJJY | -18.3 | 4.413615298 | 108 | -10.1 | 5.2 | 108 |
| Wu 2006 | XZK_AD | AD | -6 | 12.12435565 | 60 | -8 | 12.28820573 | 60 |
| Wu 2009 | XZK_AD | AD | -9 | 12.489996 | 27 | -7 | 12.489996 | 28 |
| Wu 2016 | YXQN_AD | AD | -12 | 29.64282881 | 29 | -2.6 | 27.75881662 | 29 |
| Xie 2012 | WL_AD | AD | -16.41 | 11.09834222 | 30 | -10.15 | 11.19516413 | 30 |
| Xie 2014 | YXQN_AD | AD | -14.5 | 7.560423269 | 45 | -12.6 | 8.168231143 | 45 |
| Xie 2015 | XZK_AD | AD | -22.9 | 4.232020794 | 36 | -14.9 | 5.047771786 | 36 |
| Xie 2016 | SLXMK_AD | AD | -13.16 | 3.700648592 | 120 | -8.63 | 2.984174928 | 120 |
| Xie 2017 | SLXMK_AD | AD | -11.5 | 10.1128631 | 44 | -7.5 | 9.488413988 | 46 |
| Xin 2013 | TXL_AD | AD | -0.56 | 8.837641088 | 30 | 0.88 | 9.98845834 | 30 |
| Xiong 2012 | XZK_AD | AD | -31.8 | 6.187891402 | 60 | -25 | 7.571657678 | 60 |
| Xiong 2018 | QLDX_AD | AD | -25 | 7 | 63 | -14 | 6.244997998 | 63 |
| Xu 2007 | TXL_AD | AD | -12.73 | 4.081801073 | 36 | -12.03 | 4.232457915 | 36 |
| Xu 2010 | TMGT | ZJJY | -23 | 7.211102551 | 30 | -19 | 6.557438524 | 30 |
| Xu 2012 | XKS_AD | AD | -26.2 | 8.362415919 | 83 | -22.1 | 7.715568676 | 84 |
| Xu 2013 | XZK_AD | AD | -17 | 5.632051136 | 30 | -14.8 | 6.919537557 | 30 |
| Xu 2015a | NXT_AD | AD | -9 | 6.655073253 | 90 | -2.8 | 6.90869018 | 90 |
| Xu 2015b | SLXMK_AD | AD | -28.4 | 5.703507693 | 40 | -13.8 | 6.618912297 | 40 |
| Yan 2010a | JSB_AD | AD | -14.84 | 6.297594779 | 65 | -14.887 | 6.176670543 | 50 |
| Yan 2010b | JSB_AD | AD | -12.9 | 4.092676386 | 46 | -11.2 | 4.38634244 | 46 |
| Yan 2013 | YXQN_AD | AD | -27 | 4.650806382 | 39 | -14.7 | 7.114070565 | 39 |
| Yan 2015 | XMT_AD | AD | -12.87 | 6.270318971 | 60 | -8.46 | 6.109279827 | 60 |
| Yan 2017 | QGJY_AD | AD | -23.2 | 8.185352772 | 100 | -10.5 | 7.01925922 | 100 |
| Yang 2006 | FFDS_AD | AD | -26 | 5.22045975 | 286 | -18 | 5.366292948 | 275 |
| Yang 2015 | TXL_AD | AD | -16.8 | 4.581604522 | 34 | -15.42 | 5.940227268 | 48 |
| Ye 2006 | XZK_AD | AD | -6 | 12.12435565 | 51 | -8 | 12.28820573 | 49 |
| Ye 2009 | XZK_AD | AD | -9 | 12.489996 | 28 | -7 | 12.489996 | 27 |
| You 2014 | FFDS_AD | AD | -20 | 3.605551275 | 34 | -14 | 5.196152423 | 34 |
| Yu 2005 | TXL_AD | AD | -19.4 | 5.532630477 | 27 | -8.8 | 5.164300533 | 25 |
| Yuan 2008 | SXBX_AD | AD | -23.02 | 4.864236836 | 40 | -20.32 | 5.236525566 | 40 |
| Yuan 2013 | NXT_AD | AD | -17 | 7 | 100 | -19 | 7.549834435 | 100 |
| Zhang 2006a | LWDH_AD | AD | -26 | 5.210460632 | 70 | -18 | 5.356304696 | 66 |
| Zhang 2006b | LWDH_AD | AD | -7.95 | 19.97029043 | 34 | -7.275 | 22.13453185 | 34 |
| Zhang 2011 | FFDS_AD | AD | -16.3 | 9.535722311 | 40 | -13.6 | 9.865089964 | 40 |
| Zhang 2013 | JSB_AD | AD | -11.25 | 10.45493663 | 53 | -8.44 | 9.558572069 | 53 |
| Zhang 2016a | QGJY_AD | AD | -3.5 | 3.417645388 | 47 | 0 | 3.959431777 | 44 |
| Zhang 2016b | TXL_AD | AD | -19.82 | 9.715451611 | 52 | -18.1 | 9.781104232 | 50 |
| Zhang 2016c | WL_AD | AD | -10.65 | 2.55 | 76 | -9.56 | 2.4 | 78 |
| Zhang 2016d | SXBX_AD | AD | -16.9 | 6.157921727 | 82 | -18.4 | 5.940538696 | 82 |
| Zhao 2010 | TXL_AD | AD | -13.04 | 7.577677745 | 45 | -9.66 | 8.356626113 | 45 |
| Zhao 2012 | QGJY_AD | AD | -9.2 | 4.715930449 | 40 | -5.6 | 5.08625599 | 40 |
| Zhao 2013 | XKS_AD | AD | -16 | 6.894200461 | 39 | -15.4 | 9.168969408 | 39 |
| Zhao 2016 | SSYX_AD | AD | -17.025 | 7.667626751 | 31 | -4.05 | 8.443747687 | 31 |
| Zhao 2018a | SLXMK_AD | AD | -11.68 | 10.66808793 | 47 | -6.45 | 10.84883865 | 47 |
| Zhao 2018b | XKS_AD | AD | -32.67 | 6.2540307 | 43 | -20.11 | 6.61908604 | 43 |
| Zheng 2016b | WX_AD | AD | -13.02 | 5.962994214 | 80 | -2.59 | 6.194093961 | 80 |
| Zheng 2017 | QLDX_AD | AD | -13.74 | 3.421242464 | 50 | -4.46 | 3.106235664 | 50 |
| Zhong 2017 | TMGT_AD | AD | -23.95 | 7.820479525 | 92 | -16.68 | 8.144126718 | 92 |
| Zhou 2003 | LWDH_AD | AD | -14 | 9.539392014 | 50 | -9 | 9.539392014 | 50 |
| Zhou 2006 | NHJY | AD | -17.25 | 22.68126981 | 160 | -16.95 | 17.70428973 | 120 |
| Zhou 2013 | TMGT_AD | AD | -11.4 | 6.920260111 | 48 | -11.3 | 7.28079666 | 49 |
| Zhou 2015 | ZJJY_AD | AD | -27.8 | 11.10540409 | 100 | -19.8 | 11.04852931 | 100 |
| Zhou 2018 | SSYX_AD | AD | -11.1 | 6.518435395 | 59 | -8.5 | 7.05478561 | 59 |
| Zhu 2010 | TMGT_AD | AD | -22.37 | 4.998789854 | 30 | -16.97 | 5.556077753 | 30 |
| Zhu 2012 | QJDH_AD | AD | -20.11 | 9.217000597 | 46 | -5.29 | 10.13001481 | 46 |
| Zhu 2013 | NXT_AD | AD | -24.14 | 10.81271936 | 40 | -18 | 11.05293174 | 40 |
| Zhu 2015a | XMT_AD | AD | -9 | 6.08276253 | 100 | -5 | 5.196152423 | 100 |
| Zhu 2015b | ZJJY_AD | AD | -8.13 | 7.071117309 | 110 | -2.94 | 6.353085864 | 108 |
| Zhu 2017 | SLXMK_AD | AD | -27.1 | 6.564297373 | 80 | -14.8 | 6.366317617 | 80 |
| Zhu 2018 | SLXMK | AD | -2.9 | 8.228608631 | 60 | -4.8 | 13.43465668 | 30 |
| Zhuang 2018 | TXL_AD | AD | -19.53 | 9.416724484 | 44 | -17.72 | 9.808307703 | 44 |

**Total cholesterol (TC)**

| study | Treatment | Control | Treatment Mean | Treatment SD | Treatment Sample Size | Control Mean | Control SD | Control Sample Size |
| --- | --- | --- | --- | --- | --- | --- | --- | --- |
| Wang 2010 | NXT_AD | AD | -1.07 | 0.360555128 | 35 | -0.04 | 0.435775171 | 35 |
| Zhu 2013 | NXT_AD | AD | -1.49 | 0.785047769 | 40 | -0.45 | 0.711407056 | 40 |
| Chen 2011a | QTM_AD | AD | -1.6 | 0.346410162 | 327 | -0.3 | 0.264575131 | 173 |
| Liu 2012 | QTM_AD | AD | -1.6 | 1.212435565 | 50 | -0.4 | 1.252996409 | 50 |
| Tan 2018 | QTM_AD | AD | -1.5 | 0.403608721 | 97 | -0.6 | 0.391535439 | 97 |
| He 2006 | SLXMK_AD | AD | -1 | 0.556776436 | 60 | -0.4 | 0.556776436 | 58 |
| Sun 2017b | SLXMK | AD | -1.38 | 1.099136024 | 60 | -0.32 | 1.00059982 | 30 |
| Wang 2006 | SLXMK | AD | -1.2 | 1.997498436 | 46 | -0.1 | 1.705872211 | 39 |
| Weng 2015 | SLXMK_AD | AD | -1.21 | 0.655743852 | 80 | -0.22 | 0.690217357 | 80 |
| Weng 2016 | SLXMK_AD | AD | -1.16 | 1.263605951 | 43 | -0.14 | 1.233045011 | 43 |
| Zhao 2018a | SLXMK_AD | AD | -1.33 | 1.076707946 | 47 | -0.9 | 1.101226589 | 47 |
| Cheng 2011 | TXL_AD | AD | -0.2 | 1.276714533 | 68 | -0.1 | 1.252996409 | 40 |
| Wei 2009 | TXL_AD | AD | -0.4 | 1.307669683 | 43 | -0.1 | 1.252996409 | 50 |
| Xu 2007 | TXL_AD | AD | -0.09 | 0.578878226 | 36 | -0.05 | 0.396106046 | 36 |
| Zhang 2016b | TXL_AD | AD | -2.12 | 1.542044098 | 52 | -0.55 | 1.549096511 | 50 |
| Luo 2013 | XMT_AD | AD | -0.89 | 0.825045453 | 58 | -0.18 | 0.98503807 | 58 |
| Yan 2015 | XMT_AD | AD | -1.53 | 0.625059997 | 60 | -0.91 | 0.625539767 | 60 |
| Chen 2014a | XZK_AD | AD | -0.17 | 0.160934769 | 33 | -0.03 | 0.052915026 | 32 |
| Guo 2016 | XZK_AD | AD | -2.63 | 1.238910812 | 60 | -0.1 | 1.353033629 | 60 |
| Gong 2010 | XZK_AD | AD | -0.8 | 1.352774926 | 32 | -0.8 | 1.452583905 | 30 |
| Liu 2013a | XZK_AD | AD | -1.52 | 0.828190799 | 38 | -0.16 | 1.009554357 | 39 |
| Wang 2007 | XZK_AD | AD | -0.75 | 0.842555636 | 30 | -0.03 | 0.812834547 | 30 |
| Wu 2009 | XZK_AD | AD | -0.73 | 0.595566957 | 27 | 0.28 | 0.695485442 | 28 |
| Ye 2009 | XZK_AD | AD | -0.73 | 0.595566957 | 28 | 0.28 | 0.695485442 | 27 |
| Ye 2006 | XZK_AD | AD | -0.61 | 0.811911325 | 51 | 0.01 | 0.82540899 | 49 |
| Gao 2012 | YDXNT_AD | AD | -1.05 | 0.746458304 | 57 | -0.68 | 0.763151361 | 59 |
| Wang 2012 | YDXNT_AD | AD | -0.91 | 1.198040066 | 40 | -0.23 | 1.154426264 | 37 |
| Li 2008 | XKS_AD | AD | -0.09 | 0.578878226 | 36 | -0.05 | 0.396106046 | 36 |
| Zhao 2013 | XKS_AD | AD | -1.2 | 1.55241747 | 39 | -0.1 | 1.252996409 | 39 |
| Wang 2015 | YXY_AD | AD | -2.05 | 1.00059982 | 48 | -0.49 | 1.003742995 | 48 |
| Dong 2015b | FFDS_AD | AD | -1.17 | 0.425675933 | 43 | -0.06 | 0.435775171 | 43 |
| Huang 2009 | FFDS_AD | AD | -1.07 | 0.360555128 | 33 | -0.04 | 0.435775171 | 33 |
| Yang 2006 | FFDS_AD | AD | -0.88 | 0.320468407 | 286 | -0.14 | 0.350285598 | 275 |
| Zhou 2006 | NHJY | AD | -0.98 | 1.169230516 | 52 | -0.34 | 1.450068964 | 52 |
| Fang 2019 | YXY_AD | AD | -5.6 | 3.176476035 | 100 | -4.5 | 3.377869151 | 100 |
| Hong 2014 | YXY_AD | AD | -1.57 | 0.556776436 | 65 | -0.41 | 0.556776436 | 65 |

**Triglyceride (TG)**

| Study | Treatment | Control | Treatment Mean | Treatment SD | Treatment Sample Size | Control Mean | Control SD | Control Sample Size |
| --- | --- | --- | --- | --- | --- | --- | --- | --- |
| Wang 2010 | NXT_AD | AD | -0.04 | 0.183303028 | 35 | -0.02 | 0.194679223 | 35 |
| Zhu 2013 | NXT_AD | AD | -1.12 | 0.481352262 | 40 | -0.17 | 0.466797601 | 40 |
| Chen 2011a | QTM_AD | AD | -0.8 | 0.264575131 | 327 | -0.3 | 0.346410162 | 173 |
| Liu 2012 | QTM_AD | AD | -0.9 | 0.529150262 | 50 | -0.4 | 0.435889894 | 50 |
| Tan 2018 | QTM_AD | AD | -0.9 | 0.407062649 | 97 | 0.02 | 0.400374824 | 97 |
| He 2006 | SLXMK_AD | AD | -0.4 | 0.608276253 | 60 | -0.3 | 0.458257569 | 58 |
| Sun 2017b | SLXMK | AD | -0.53 | 0.533572863 | 60 | -0.23 | 0.560446251 | 30 |
| Wang 2006 | SLXMK | AD | -0.4 | 0.781024968 | 46 | -0.2 | 0.953939201 | 39 |
| Weng 2015 | SLXMK_AD | AD | -0.41 | 0.6244998 | 80 | -0.09 | 0.780768852 | 80 |
| Weng 2016 | SLXMK_AD | AD | -0.47 | 0.521152569 | 43 | -0.11 | 0.54064776 | 43 |
| Zhao 2018a | SLXMK_AD | AD | -0.69 | 0.848704896 | 47 | -0.38 | 0.998599019 | 47 |
| Cheng 2011 | TXL_AD | AD | -0.1 | 0.4 | 68 | -0.1 | 0.556776436 | 40 |
| Luo 2013 | XMT_AD | AD | -0.26 | 0.320468407 | 58 | -0.06 | 0.280535203 | 58 |
| Yan 2015 | XMT_AD | AD | -0.64 | 0.673572565 | 60 | -0.25 | 0.605557594 | 60 |
| Guo 2016 | XZK_AD | AD | -0.61 | 0.781728853 | 60 | 0.03 | 0.520480547 | 60 |
| Liu 2013a | XZK_AD | AD | -0.87 | 0.281602557 | 38 | 0.08 | 0.338673884 | 39 |
| Wang 2007 | XZK_AD | AD | -0.87 | 0.681469001 | 30 | -0.03 | 0.648151217 | 30 |
| Ye 2006 | XZK_AD | AD | -0.11 | 0.60506198 | 51 | 0.06 | 0.605557594 | 49 |
| Gao 2012 | YDXNT_AD | AD | -1.1 | 0.90066642 | 57 | -0.75 | 0.973498844 | 59 |
| Wang 2012 | YDXNT_AD | AD | -0.51 | 1.316396597 | 40 | -0.06 | 1.396316583 | 37 |
| Zhao 2013 | XKS_AD | AD | -0.6 | 0.781024968 | 39 | 0 | 1.153256259 | 39 |
| Wang 2015 | YXY_AD | AD | -0.96 | 0.347706773 | 48 | -0.17 | 0.365923489 | 48 |
| Dong 2015b | FFDS_AD | AD | -0.03 | 0.196723156 | 43 | -0.04 | 0.17691806 | 43 |
| Huang 2009 | FFDS_AD | AD | -0.04 | 0.183303028 | 33 | -0.02 | 0.194679223 | 33 |
| Yang 2006 | FFDS_AD | AD | -0.66 | 0.14 | 286 | -0.06 | 0.14106736 | 275 |
| Zhou 2006 | NHJY | AD | -0.51 | 0.979642792 | 52 | -0.14 | 1.100136355 | 52 |
| Ou Yang 2002 | QJDH_AD | AD | -0.5 | 0.692820323 | 34 | -0.2 | 0.9 | 34 |
| Fang 2019 | YXY_AD | AD | -1.1 | 0.529150262 | 100 | -0.4 | 0.655743852 | 100 |
| Hong 2014 | YXY_AD | AD | -0.07 | 0.360555128 | 65 | -0.03 | 0.793725393 | 65 |

**Adverse effects (AE)**

| Study | Treatment | Control | Treatment Responders | Treatment Sample Size | Control Responders | Control Sample Size |
| --- | --- | --- | --- | --- | --- | --- |
| Chai 2007 | FFDS_AD | AD | 4 | 50 | 5 | 50 |
| Chen 2011b | SXBX_AD | AD | 5 | 65 | 6 | 65 |
| Chen 2013d | SXBX_AD | AD | 1 | 64 | 8 | 64 |
| Chen 2015 | WL_AD | AD | 7 | 48 | 10 | 48 |
| Dai 2006 | NHJY | AD | 2 | 33 | 7 | 30 |
| Fang 2012 | ZJJY | AD | 4 | 142 | 13 | 142 |
| Fu 2009a | JSB_AD | AD | 6 | 35 | 3 | 30 |
| Fu 2009b | SLXMK_AD | AD | 1 | 35 | 0 | 35 |
| Gong 2010 | XZK_AD | AD | 3 | 32 | 2 | 30 |
| Gui 2017 | YDXNT_AD | AD | 7 | 101 | 23 | 100 |
| He 2006 | SLXMK_AD | AD | 2 | 60 | 1 | 58 |
| He 2012 | ZJJY | AD | 3 | 60 | 6 | 60 |
| Hou 2010 | ZJJY | AD | 2 | 48 | 4 | 42 |
| Huang 2009 | FFDS_AD | AD | 2 | 33 | 0 | 33 |
| Ji 2011 | YXQN_AD | AD | 2 | 80 | 0 | 80 |
| Jiang 2011 | SLXMK_AD | AD | 3 | 52 | 7 | 50 |
| Li 2003 | TXL_AD | AD | 6 | 44 | 0 | 42 |
| Liu 2014 | TMGT_AD | AD | 6 | 63 | 5 | 64 |
| Liu 2015a | QGJY_AD | AD | 5 | 89 | 4 | 89 |
| Liu 2016a | YXQN_AD | AD | 4 | 45 | 9 | 45 |
| Liu 2017 | NXT_AD | AD | 1 | 60 | 3 | 60 |
| Luo 2013 | XMT_AD | AD | 2 | 58 | 8 | 58 |
| Mao 2017 | NXT_AD | AD | 6 | 81 | 17 | 80 |
| Mo 2009 | TXL_AD | AD | 9 | 120 | 0 | 120 |
| Peng 2018 | WX_AD | AD | 3 | 60 | 5 | 60 |
| Shen 2007 | NXT_AD | AD | 1 | 30 | 1 | 30 |
| Shi 2015 | YXQN_AD | AD | 1 | 199 | 1 | 199 |
| Shi 2016 | YXQN_AD | AD | 2 | 42 | 2 | 41 |
| Sun 2015 | YXY_AD | AD | 0 | 35 | 1 | 35 |
| Wang 2007 | XZK_AD | AD | 2 | 30 | 0 | 30 |
| Wang 2010 | NXT_AD | AD | 2 | 35 | 0 | 35 |
| Wei 2014 | TMGT_AD | TMGT | 4 | 64 | 5 | 64 |
| Weng 2016 | SLXMK_AD | AD | 2 | 43 | 2 | 43 |
| Xie 2017 | SLXMK_AD | AD | 1 | 44 | 1 | 46 |
| Xiong 2018 | QLDX_AD | AD | 8 | 63 | 9 | 63 |
| Xu 2012 | XKS_AD | AD | 8 | 83 | 8 | 84 |
| Xu 2015b | SLXMK_AD | AD | 1 | 40 | 1 | 40 |
| Yan 2013 | YXQN_AD | AD | 1 | 39 | 1 | 39 |
| Yang 2015 | TXL_AD | AD | 1 | 33 | 0 | 46 |
| Zhang 2016b | TXL_AD | AD | 3 | 52 | 2 | 50 |
| Zhao 2010 | TXL_AD | AD | 2 | 45 | 1 | 45 |
| Zheng 2016b | WX_AD | AD | 6 | 80 | 8 | 80 |
| Zhong 2017 | TMGT_AD | AD | 6 | 92 | 4 | 92 |
| Zhou 2018 | SSYX_AD | AD | 5 | 59 | 13 | 59 |
| Zhu 2015b | ZJJY_AD | AD | 12 | 110 | 7 | 108 |
| Zhuang 2018 | TXL_AD | AD | 3 | 44 | 2 | 44 |
